# Synthesis and Structure Optimization of Star Copolymers as Tunable Macromolecular Carriers for Minimal Immunogen Vaccine Delivery

**DOI:** 10.1021/acs.bioconjchem.4c00273

**Published:** 2024-07-31

**Authors:** Gabriela Mixová, Eva Tihlaříková, Yaling Zhu, Lucie Schindler, Ladislav Androvič, Lucie Kracíková, Eliška Hrdá, Bedřich Porsch, Michal Pechar, Christopher M. Garliss, David Wilson, Hugh C. Welles, Jake Holechek, Qiuyin Ren, Geoffrey M. Lynn, Vilém Neděla, Richard Laga

**Affiliations:** † Institute of Macromolecular Chemistry, Czech Academy of Sciences, Heyrovského nám. 2, Prague 162 06, Czech Republic; ‡ Institute of Scientific Instruments, Czech Academy of Sciences, Královopolská 147, Brno 612 64, Czech Republic; § Barinthus Biotherapeutics North America, Inc. (formerly Avidea Technologies, Inc.), 20400 Century Boulevard, Germantown, Maryland 20874, United States; ∥ Vaccine Research Center, National Institutes of Health, Rockville, Maryland 20892, United States

## Abstract

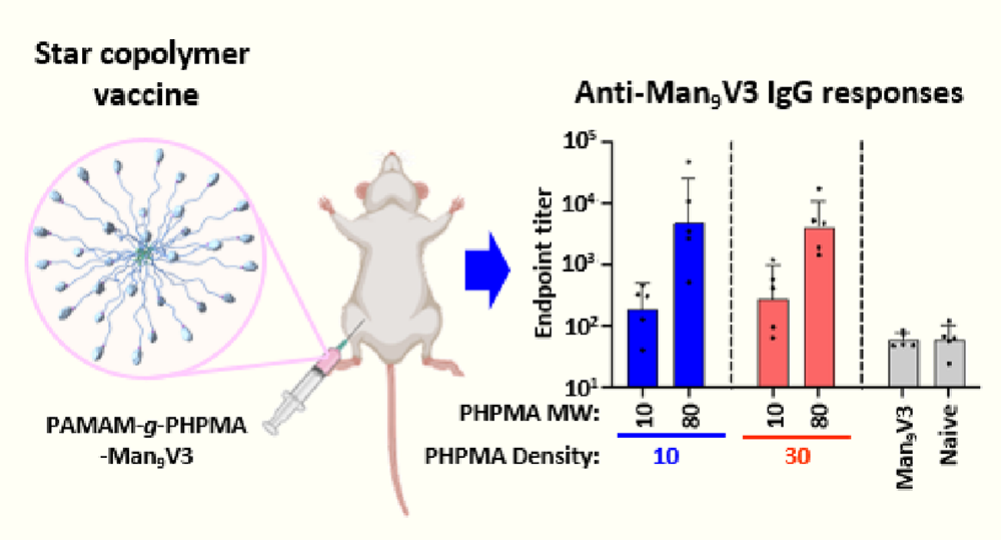

Minimal immunogen vaccines are being developed to focus antibody responses against otherwise challenging targets, including human immunodeficiency virus (HIV), but multimerization of the minimal peptide immunogen on a carrier platform is required for activity. Star copolymers comprising multiple hydrophilic polymer chains (“arms”) radiating from a central dendrimer unit (“core”) were recently reported to be an effective platform for arraying minimal immunogens for inducing antibody responses in mice and primates. However, the impact of different parameters of the star copolymer (e.g., minimal immunogen density and hydrodynamic size) on antibody responses and the optimal synthetic route for controlling those parameters remains to be fully explored. We synthesized a library of star copolymers composed of poly[*N*-(2-hydroxypropyl)methacrylamide] hydrophilic arms extending from poly(amidoamine) dendrimer cores with the aim of identifying the optimal composition for use as minimal immunogen vaccines. Our results show that the length of the polymer arms has a crucial impact on the star copolymer hydrodynamic size and is precisely tunable over a range of 20–50 nm diameter, while the dendrimer generation affects the maximum number of arms (and therefore minimal immunogens) that can be attached to the surface of the dendrimer. In addition, high-resolution images of selected star copolymer taken by a custom-modified environmental scanning electron microscope enabled the acquisition of high-resolution images, providing new insights into the star copolymer structure. Finally, *in vivo* studies assessing a star copolymer vaccine comprising an HIV minimal immunogen showed the criticality of polymer arm length in promoting antibody responses and highlighting the importance of composition tunability to yield the desired biological effect.

## Introduction

Most modern vaccines for infectious disease prevention use full-length protein immunogens derived from a pathogenic organism (e.g., hemagglutinin from influenza virus or SARS-CoV-2 spike protein from corona virus) to induce antibodies that can prevent infection.^1−3^ While this strategy has been highly effective in preventing many diseases, full-length proteins have two key limitations. First, use of full-length proteins that are typically composed of hundreds of amino acids can lead to antibody responses against highly variable sites of the pathogen that distracts antibody responses away from conserved sites needed for efficacy.^4^ Second, antibodies generated against certain sites of full-length protein have been observed to exacerbate disease after infection in a process called antibody-mediated disease enhancement (ADE).^5−7^

An emerging strategy to overcome these limitations is to use only an active part of the full-length protein, referred to as a minimal immunogen, which allows antibody responses to be focused against key sites while avoiding those that could lead to ADE.^8,9^ However, minimal immunogens, in isolation, are poorly immunogenic.^10^ This is due to their relatively low molecular weight (<10 kg·mol^–1^) resulting in rapid clearance and poor drainage to lymph nodes (LNs)^11^ and the inability of soluble, minimal immunogens to sufficiently cross-link B cell receptors needed for antibody induction.^12^ To address these challenges, nanoparticles and other macromolecular carriers have been introduced to increase size as a means for reducing elimination half-life and scaffolding the immunogen to promote B cell receptor cross-linking.^13−15^

Some of the most advanced minimal immunogen vaccines in development have relied on recombinant nanoparticles that fuse the minimal immunogen to a structural protein that self-assembles into a multimer displaying multiple copies of the minimal immunogen.^16^ Approaches under development include those using bacteriophages,^17^ keyhole limpet hemocyanin (KLH, a naturally occurring multimeric protein nanoparticle),^18^ ferritin nanoparticles, and various virus-like particles.^19,20^ While these recombinant approaches have shown promise, certain minimal immunogens contain post-translation modifications (PTMs), including glycosylation sites key for antibody recognition that cannot be readily encoded using recombinant approaches;^21^ additionally, the number and density of immunogens that can affect antibody responses cannot be readily controlled.^22,23^

While various synthetic nanocarriers based on lipids, amphiphilic polymers, and peptides or inorganic metal-based nanoparticles have been introduced for delivering minimal peptide immunogens, most were developed for delivering full-length protein or inducing T cell responses and are often limited in controlling minimal immunogen density and orientation in a stable array.^24−26^

As an alternative, fully synthetic star copolymer-based vaccines comprising minimal immunogens linked to hydrophilic polymer arms radiating from dendrimer cores provide the potential advantages that minimal immunogen density, orientation, and scaffold size can be precisely tuned to modulate antibody responses. Earlier work has established the utility of star copolymers as tunable platforms for a range of medicinal applications including delivery of chemotherapeutics for cancer treatment as well as prevention or treatment of genitourinary tract infections, providing proof of concept for use in humans.^27−32^ More recently, star copolymer-based minimal immunogen vaccines were shown to be safe for administration to mice and primates for inducing neutralizing antibody responses; however, the impact of various structural parameters (minimal immunogen density and hydrodynamic diameter) on antibody responses and the synthetic approach to optimize such parameters needs further investigation.^33^ Indeed, ligand density, linker length, and hydrodynamic size have been shown to impact antibody responses with other platforms.^12−14^ Furthermore, optimizing these parameters for glycosylated minimal immunogens, which are notoriously poorly immunogenic may be even more critical to ensuring effective antibody responses.^34^

Therefore, the objectives of this work were to investigate how various parameters of star copolymer vaccines (polymer arm length, minimal peptide immunogen density, etc.) affect star copolymer physicochemical properties (e.g., hydrodynamic size) and capacity for inducing antibody responses against a glycosylated minimal immunogen (“Man9 V3”) derived from human immunodeficiency virus (HIV).^35^ A custom-modified environmental scanning electron microscope (ESEM) equipped with a scanning transmission electron microscopy (STEM) detector was used to provide unique ultrahigh-resolution images of selected star copolymers that document their size and shape in the unsolvated state. After optimizing synthetic conditions for controlling polymer arm loading and star copolymer vaccine size, we examined how minimal immunogen density and hydrodynamic size impact antibody responses. The results show how the synthetic approach can be used to control the minimal peptide immunogen density and hydrodynamic size of star copolymer vaccines for promoting antibody responses.

## Results and Discussion

### Synthesis and Characterization of Star Copolymers

**Scheme 1:**
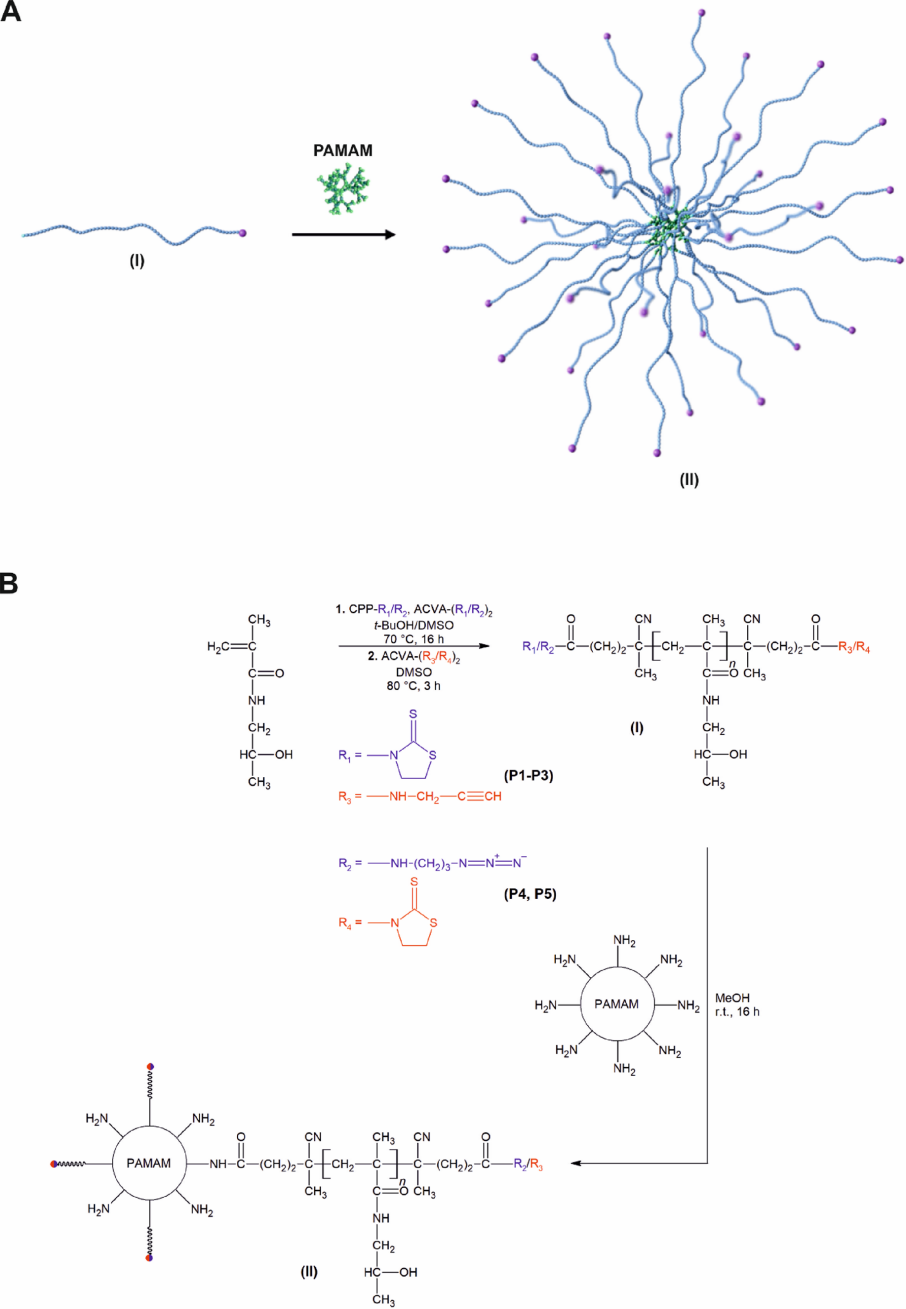
Cartoon Depiction (A) and Reaction Scheme (B) for the Synthesis of Star Copolymers (II) Composed of the PAMAM Dendrimer Core and Heterobifunctional HPMA-Based Arms (I)

The preparation of the star copolymer was performed in two synthetic steps, including the synthesis of heterobifunctional polymer arms, followed by conjugation of the arms to the dendrimer cores (for the reaction scheme, see Scheme 1).

The polymer arms **P1**–**P5** were synthesized by the reversible addition–fragmentation chain-transfer (RAFT) polymerization of HPMA in the presence of different molar amounts of the thiazolidine-2-thione (TT)- (for polymers **P1–P3**) or azide group (N_3_)-functionalized (for polymers **P4** and **P5**) chain-transfer agent and initiator. The polymers of number-average molecular weights (*M*_n_) of ∼10–80 kg·mol^**–**1^ were distinguished by a narrow molecular weight distribution (*Đ* ≤ 1.1, *Đ* = *M*_w_/*M*_n_) and high functionalities (*f* ≥ 0.8, *f* is defined as the number of functional end groups per polymer chain calculated as the ratio between *M*_n_ obtained from the size exclusion chromatography (SEC) measurement and *M*_n_ calculated from the end group analysis) of either TT or azide end groups. The dithiobenzoate (DTB) groups of the heterobifunctional polymers were subsequently replaced with propargyl (for polymers **P1–P3**) or TT groups (for polymers **P4** and **P5**) by the homolytic reaction with a high molar excess of the functionalized azoinitiators to introduce the reactive moieties to the ends of the polymer arms for the biorthogonal attachment of the peptide vaccine. No significant changes in *M*_n_ and *Đ* nor a decrease in the reactive groups at opposite ends of the chains were observed in this substitution reaction. The characteristics for heterobifunctional polymers **P1–P5** are summarized in Table 1, and their SEC profiles are depicted in Figure S2.

**Table 1:** Characteristics of the Heterobifunctional PHPMA Polymer Arms with TT Groups at One Side and Propargyl (**P1**–**P3**) or Azide Groups (**P4** and **P5**) on the Other Side of the Polymer Chain

**polymer arm**	**[M]**_**0**_**/[CTA]**_**0**_**/[I]**_**0**_^i^	***M*_n_**^ii^ [kg·mol^–1^]	***Đ***iii	***R*_g_**^iv^**[nm]**	*f***(TT)**^v^
**P1**	135:1:0.5	9.8	1.03	n.d.	0.97
**P2**	200:1:0.5	15.8	1.08	3.9	0.91
**P3**	698:1:0.5	41.1	1.05	6.1	0.86
**P4**	65:1:0.5	8.6	1.05	n.d.	0.92
**P5**	700:1:0.5	71.3	1.10	8.0	0.81

i Ratio of the molar concentrations of monomer (M), chain transfer agent (CTA), and initiator (I) in the polymerization feed.

ii Number-average molecular weight of the polymer arm determined by SEC.

iii Polymer arm dispersity defined as the ratio of weight-average (*M*_w_) to number-average (*M*_n_) molecular weight determined by SEC.

iv Radius of gyration of the polymer arm determined by SEC.

v Polymer arm functionality defined as the average number of TT groups per polymer chain.

The heterobifunctional polymer arms **P1–P3** with TT and propargyl end groups were then grafted onto PAMAM dendrimers of different generations, varying in molecular weight and number of surface ∼NH_2_ groups (for PAMAM dendrimer characteristics, see Table S1), using three different molar ratios of the PAMAM surface ∼NH_2_ groups to the TT terminal group on the polymer arms (1:1, 2:1, and 3:1). The conjugation reaction was carried out in dry methanol to prevent competitive hydrolysis of the TT groups and hence lower conjugation recovery. Based on the reaction conditions and the composition of the reactants, the resulting product was a mixture of star copolymers of various physicochemical parameters (**S1**–**S35**) and unreacted heterobifunctional polymer. Although the resulting product consisted of a binary mixture of two polymers, suitably selected chromatographic conditions in combination with light scattering (LS) and refractive index (RI) detectors (for details, see Materials and Methods section) allowed precise characterization of both types of materials without the need for purification. Only the star copolymer **S17**, which was further used for covalent coupling with the electron microscopy (EM) contrast label and star copolymers **S28**–**S31**, which were further used for minimal peptide immunogen binding, were purified from unreacted linear polymer using centrifugal filter units (for SEC chromatograms of the star copolymer **S17** before and after the purification, see Figure S1). All star copolymers, irrespective of the method of preparation, exhibited a narrow distribution of molecular weights (*Đ* ≤ 1.35), suggesting the preparation of defined materials. The influence of polymer arm length, dendrimer generation, and reactant functional group ratio on the size, molecular weight, morphology, and yield of the star copolymer was investigated in detail (for star copolymer characteristics, see Table 2).

**Table 2:** Characteristics of Star Copolymers **S1**–**S26** Composed of Polymer Arms **P1**–**P3** Extending from PAMAM Dendrimers G3–G5, Prepared at Three Different Molar Ratios (1:1, 2:1, and 3:1) of the PAMAM Surface ~NH_2_ Groups to the ~TT Terminal Group on the Polymer Arms

**star copolymer**	**polymer arm**	**PAMAM generation**	*n***(~NH**_**2**_**)/***n***(~TT)**^i^	***M*_n_**^ii^ [kg·mol^–1^]	***Đ***iii	***N***^iv^**(polymer arms)**	**yield**^v^**[%]**	***R*_g_**^vi^**[nm]**
**S1**	P1	G3	1:1	246.6	1.12	24	48.7	9.6
**S2**	P1	G3	2:1	215.6	1.19	21	73.9	10.3
**S3**	P1	G3	3:1	157.0	1.17	15	76.7	9.5
**S4**	P1	G4	1:1	311.6	1.19	30	46.1	9.6
**S5**	P1	G4	2:1	242.3	1.30	23	70.4	10.8
**S6**	P1	G4	3:1	182.8	1.27	17	72.9	9.2
**S7**	P1	G5	1:1	405.1	1.21	38	40.7	10.2
**S8**	P1	G5	2:1	332.3	1.22	31	63.0	10.2
**S9**	P1	G5	3:1	235.3	1.31	21	71.9	9.9
**S10**	P2	G3	1:1	488.2	1.16	30	48.9	12.6
**S11**	P2	G3	2:1	336.5	1.22	20	68.8	13.0
**S12**	P2	G3	3:1	204.7	1.14	16	70.7	10.4
**S13**	P2	G4	1:1	452.6	1.22	28	43.2	12.5
**S14**	P2	G4	2:1	344.5	1.30	21	67.4	13.1
**S15**	P2	G4	3:1	251.0	1.29	15	69.2	12.1
**S16**	P2	G5	1:1	589.2	1.18	36	28.5	12.6
**S17**	P2	G5	2:1	501.7	1.35	30	62.4	14.5
**S18**	P2	G5	3:1	364.7	1.35	21	67.0	13.6
**S19**	P3	G3	1:1	709.9	1.03	17	52.1	17.0
**S20**	P3	G3	3:1	425.7	1.06	10	64.9	15.0
**S21**	P3	G4	1:1	889.4	1.06	21	65.6	18.4
**S22**	P3	G4	2:1	649.5	1.07	15	60.5	17.6
**S23**	P3	G4	3:1	500.5	1.08	12	64.1	16.1
**S24**	P3	G5	1:1	1272.0	1.04	30	34.8	18.6
**S25**	P3	G5	2:1	1045.0	1.06	25	53.9	19.0
**S26**	P3	G5	3:1	776.6	1.08	18	57.9	17.0

i Molar ratio of PAMAM∼NH_2_ groups to PHPMA∼TT groups in the reaction mixture.

ii Number-average molecular weight of the star copolymer determined by SEC.

iii Star copolymer dispersity defined as the ratio of weight-average (*M*_w_) to number-average (*M*_n_) molecular weight determined by SEC.

iv Number of polymer arms attached to the PAMAM dendrimer core evaluated by SEC.

v Yield of the conjugation reaction evaluated by SEC.

vi Radius of gyration of the star copolymer determined by SEC.

### Polymer Arm Length Is a Key Determinant of Star Copolymer Size

The size of the carrier used to deliver minimal immunogens has been shown to be a key factor for influencing immune responses. Therefore, we sought to investigate whether polymer arm length could be used to modulate the star copolymer hydrodynamic size.

**Figure 1:**
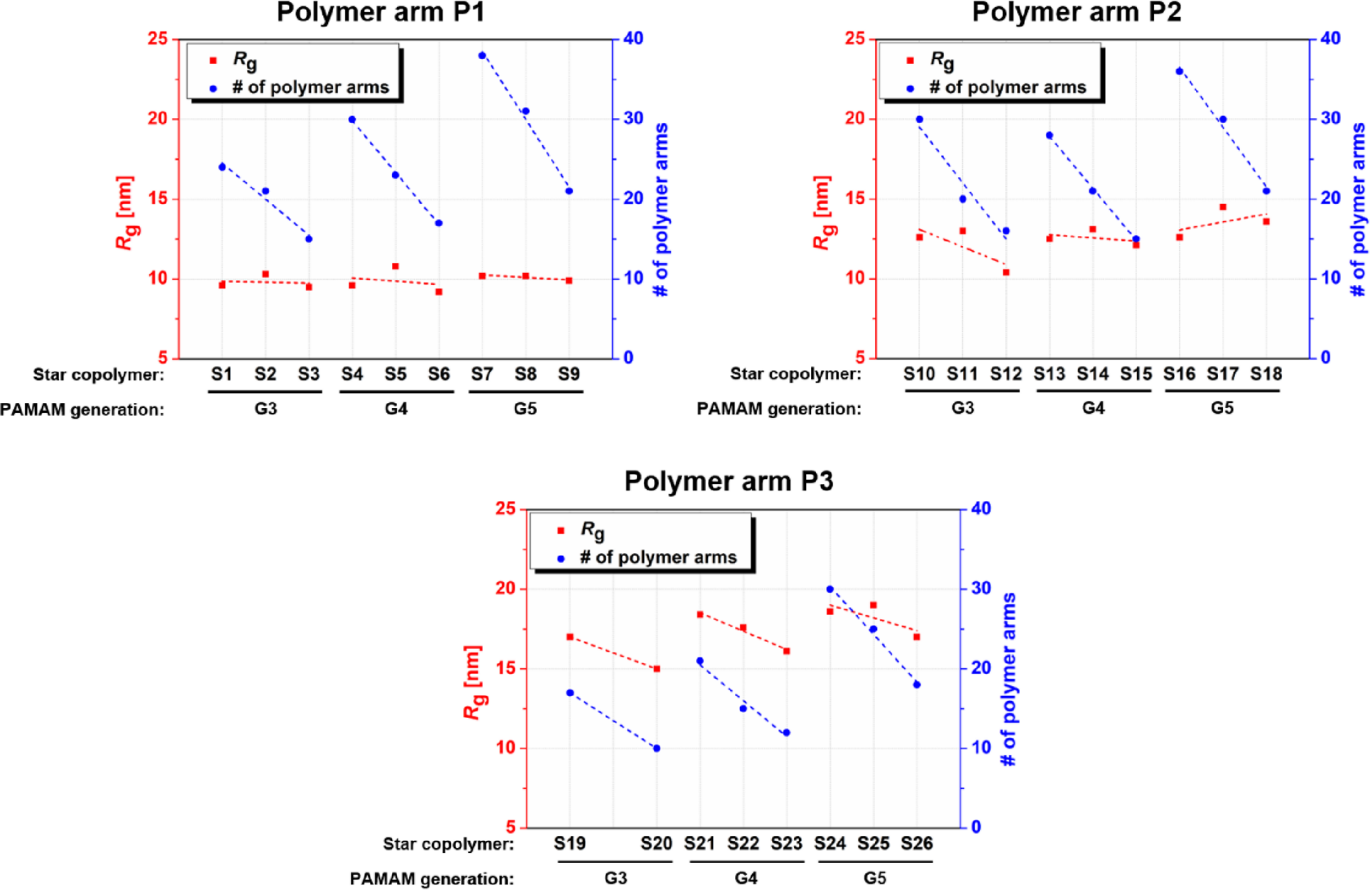
Influence of PAMAM dendrimer generation and molar ratio of the PAMAM surface. ~NH_2_ groups to the ~TT terminal group on the polymer arms on the gyration radius (*R*_g_) and number (#) of polymer arms of star copolymers.

Our results showed that the length of the polymer arm was found to play a key role in designing star copolymers of a certain size and the number of grafts that can be linked to the dendrimer core. In general, the gyration radius (*R*_g_) and the number-average molecular weight (*M*_n_) of the star copolymers increase with an increasing *M*_n_ of the polymer arm using a dendrimer of the same generation and the same molar ratio of reactants (see Table 2 and Figure 1). For example, the star copolymer **S8** consisting of the arms of polymer **P1** (9.8 kg·mol^–1^) has *R*_g_ and *M*_n_ values of 10.2 nm and 332.3 kg·mol^–1^, respectively; *R*_g_ and *M*_n_ of the star copolymer **S17** with the **P2** arms (15.8 kg·mol^–1^) are 14.5 nm and 501.7 kg·mol^–1^; and the star copolymer **S26** formed by the **P3** arms (41.1 kg·mol^–1^) has *R*_g_ and *M*_n_ of 19.0 nm and 1045.0 kg·mol^–1^. These data show that the star copolymer size can be precisely tuned by controlling the polymer arm *M*_n_.

Perhaps not unexpectedly, the number of arms that can be attached to the dendrimer core (*N*) decreases with increasing polymer arm *M*_n_ (see Table 2 and Figure 1). Accordingly, for star copolymers **S4**, **S13**, and **S22** comprising polymer arms **P1**, **P2**, and **P3** with increasing molecular weight, the number of polymer arms attached (*N*) is 30, 28, and 21. As size and minimal immunogen density can impact biological activity, these competing factors must therefore be balanced in the design of star copolymers for use as minimal vaccines.

### Dendrimer Core Generation Impacts Arm Density but not Star Copolymer Size

The dendrimer core generation (G3–G5) was another parameter assessed for its impact on size, molecular weight, and number of polymer arms attached. Our results showed that the *R*_g_ values of star copolymers were almost independent of the dendrimer generation, while the number of attached polymer arms and hence the *M*_n_ of the star copolymers increased linearly (see Table 2 and Figure 1). For example, attaching polymer arms **P1** to PAMAM, G3 resulted in a 9.6 nm star copolymer **S1** with 24 arms, whereas the star copolymer **S4** composed of PAMAM, G4 had a *R*_g_ of 9.6 nm and 30 arms, and the star copolymer **S7** measured 10.2 nm and contained 38 arms. These results suggest that the PAMAM dendrimer generation (i.e., degree of dendrimer branching) affects the density of the arms that can be attached, but minimally impact the size of the star copolymer, and that increasing the dendrimer generation can be used as a means for accommodating higher densities of polymers chains. Given the higher density of arm loading that can be achieved with higher generations of PAMAM dendrimers, G5 PAMAM dendrimers were selected as the preferred core for further evaluation as vaccines later. Though, as the studies herein only evaluated G3-G5 dendrimers, further assessment may be warranted to understand if the observed trends apply to lower and higher dendrimer generations (<G3, > G5).

### Impact of Polymer to Dendrimer Molar Ratio on Star Copolymer Size and Yield

We also assessed whether the synthetic scheme had an impact on the star copolymer properties. Our results showed that star copolymer size and yield are impacted by the reactant molar ratio. The data showed that both the *N* and the *M*_n_ of the star copolymers decreased linearly with increasing molar ratio of PAMAM **~**NH_2_ groups to PHPMA **~**TT groups, regardless of the polymer arm length and PAMAM generation used (see Table 2 and Figure 1). For example, star copolymers **S13**, **S14**, and **S15** were synthesized by mixing PAMAM, G4 with polymer arm **P2** at functional group ratios (*n*(~NH_2_)/*n*(~TT)) of 1:1, 2:1, and 3:1, respectively. The resulting star copolymer **S13** had an *M*_n_ of 452.6 kg·mol^–1^ and 28 polymer arms; the copolymer **S14** had 344.5 kg·mol^–1^ and 21 polymer arms; and the copolymer **S15** had 251.0 kg·mol^–1^ and 15 polymer arms. This suggests that at lower ratios (*n*(~NH_2_)/*n*(~TT)) when there are fewer amines available on the dendrimer core, there is steric hindrance that results in competition for binding, which is also consistent observed yields of 43, 67, and 69% for **S13**, **S14**, and **S15,** respectively. Notably, increasing the *n*(~NH_2_)/*n*(~TT) ratio has minimal to no impact on the size of the resulting star copolymers, with **S13**, **S14**, and **S15**, all having *R*_g_ values between ∼12 and 13 nm. This can be partly attributed to the low dependence of the star copolymer size on the number of polymer arms and partly to the different sensitivity of the characterization technique (static light scattering) to changes in the monitored quantities (while the weight-average molecular weight (*M*_w_), according to the Rayleigh’s approximation for the spherical particle, increases linearly with the scattering intensity (*I*_LS_), the diameter is proportional to the sixth root of the *I*_LS_). Based on these data, the reaction scheme using a 2:1 ratio of core and polymer chain functional groups, *n*(~NH_2_)/*n*(~TT), was selected as the preferred route as it affords star copolymers with high polymer arm density at an acceptable yield.

### Ultrahigh-Resolution Imaging of Star Copolymers

**Figure 2:**
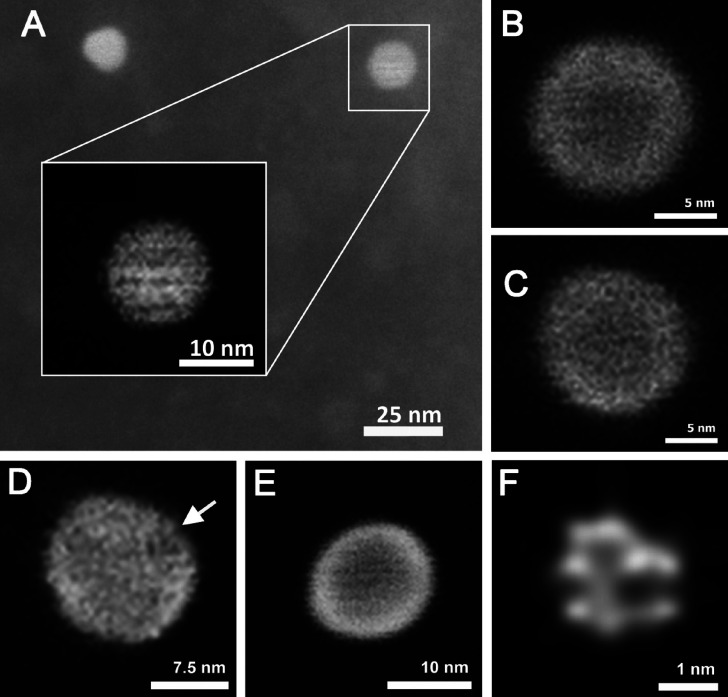
Examples of ultrahigh-resolution images of star copolymers **S17**: (A–E) STEM analysis of star copolymers. Indication of the inner arrangement is viewed. The differences between the images are due to the different distribution of Au_11_ nanoparticles in the sample. (F) Cluster of 8 free Au-based nanoparticles, each with a diameter of 0.8 nm, corresponding to the Au_11_.

To our knowledge, the size and shape of star copolymer vaccines have not been assessed in detail. Therefore, ultrahigh-resolution images of selected star copolymers were provided to document their size and shape. For these purposes, the purified star copolymer **S17** was chosen as a representative carrier with satisfactory size (*R*_g_ = 29 nm) and a high number of polymer arms (*N* = 30). To increase the signal-to-noise ratio and improve the contrast of the image from the STEM detector, the copolymer was labeled with Au-based nanoparticles (mono-sulfo-NHS-undecagold, Au_11_). In this work, the Au_11_ nanoparticles were attached to either (i) the surface of the PAMAM core, (ii) the end groups of the PHPMA arms, or (iii) both the PAMAM core and the ends of the PHPMA arms. We found that the best imaging was provided by a copolymer that was labeled only at the ends of the polymer arms, with the contrast of the micrographs also being affected by the number of attached Au_11_ nanoparticles. From the group of star copolymers decorated with approximately 30, 15, and 5 contrast agent molecules, the one with the smallest number of Au_11_ nanoparticles (5) proved to be the most suitable for STEM analysis. Several examples of micrographs of star copolymer **S17** containing ∼5 Au_11_ nanoparticles at the end of its PHPMA arms are shown in Figure 2.

The observed local decrease in the intensity of the detected signal (or the contrast, respectively) in the analyzed sample (Figure 2A–E) can be attributed to the absence of Au_11_ nanoparticles on PHPMA arms (only ∼17% of the arms are modified with Au_11_) or the spatial arrangement of individual PHPMA arms (some ends of the arms may be hidden inside the polymer coils). In Figure 2 D (white arrow), it can be seen – due to in situ freeze-drying sample preparation – that there is local change of shape or length of the polymer arms. In contrast, a sample with an intact structure and a homogeneous distribution of arms is shown in Figure 2E. The more contrasting area around the star copolymer core (compared to the outer edges) may indicate that the dense dendrimer core (∼6 nm in diameter for G5) is surrounded by expanded chains of hydrophilic polymer arms (Figure 2B,C). Although the obtained micrographs of star copolymers are two-dimensional projections into the *X* and *Y* planes, based on changes in intensity in image contrast, star copolymers can be described as spatially symmetric structures of approximately a spherical shape. The most frequently observed star copolymers of a spherical shape with a typical radius of about 7.5 nm are depicted in Figure 2A–D. The difference between the radius of the samples measured by EM and dynamic light scattering (DLS) (*R*_h_ ∼ 15 nm) is about 50%. This can be attributed to the different measurement conditions of the two methods; while in the case of electron microscopy, the sample is measured in a completely dried state (using the freeze-drying method), with DLS, the sample is analyzed in a fully solvated state (aqueous solution), which causes the expansion (swelling) of the arms. In addition, the DLS provides a z-average size that is strongly influenced by the presence (even of low populations) of large particles, while the resulting particle size from the EM measurement is calculated by arithmetic averaging, giving the number-average size. Spatially asymmetric formations (Figure 2E) were observed very rarely.

Since the resolution required for convincing imaging of samples is at the limit of the possibilities of the microscope used, the obtained micrographs had to be modified by postprocessing methods. Brightness and contrast were optimized, in particular. The effect of micrograph modification can be seen in Figure 2A, where the background of the micrograph is the original raw micrograph, whereas the postprocessed micrograph is depicted in a white frame with bar. During the detailed analysis of the sample, it was possible to find also an area in which a cluster of free (unbound or released) Au_11_ nanoparticles can be seen (Figure 2 F). The measured size of Au nanoparticles in the cluster (0.6–0.8 nm) corresponds to the size of the undecagold (Au_11_) contrast agent, which is reported by the manufacturer (Nanoprobes Inc.). Star copolymer micrographs confirm a notably uneven surface structure (Figure 2A–E), which is consistent with the intended application of these materials as carriers of minimal peptide immunogens mimicking viruses.

### Synthesis and Characterization of Star Copolymer Vaccines

After having optimized a synthetic route for modulating polymer arm density and particle size, we next sought to investigate how these parameters impact biological activity of a star copolymer vaccine delivering a representative minimal immunogen consisting of a glycopeptide derived from the V3 loop of HIV Envelope protein, referred to as Man_9_V3.

**Scheme 2:**
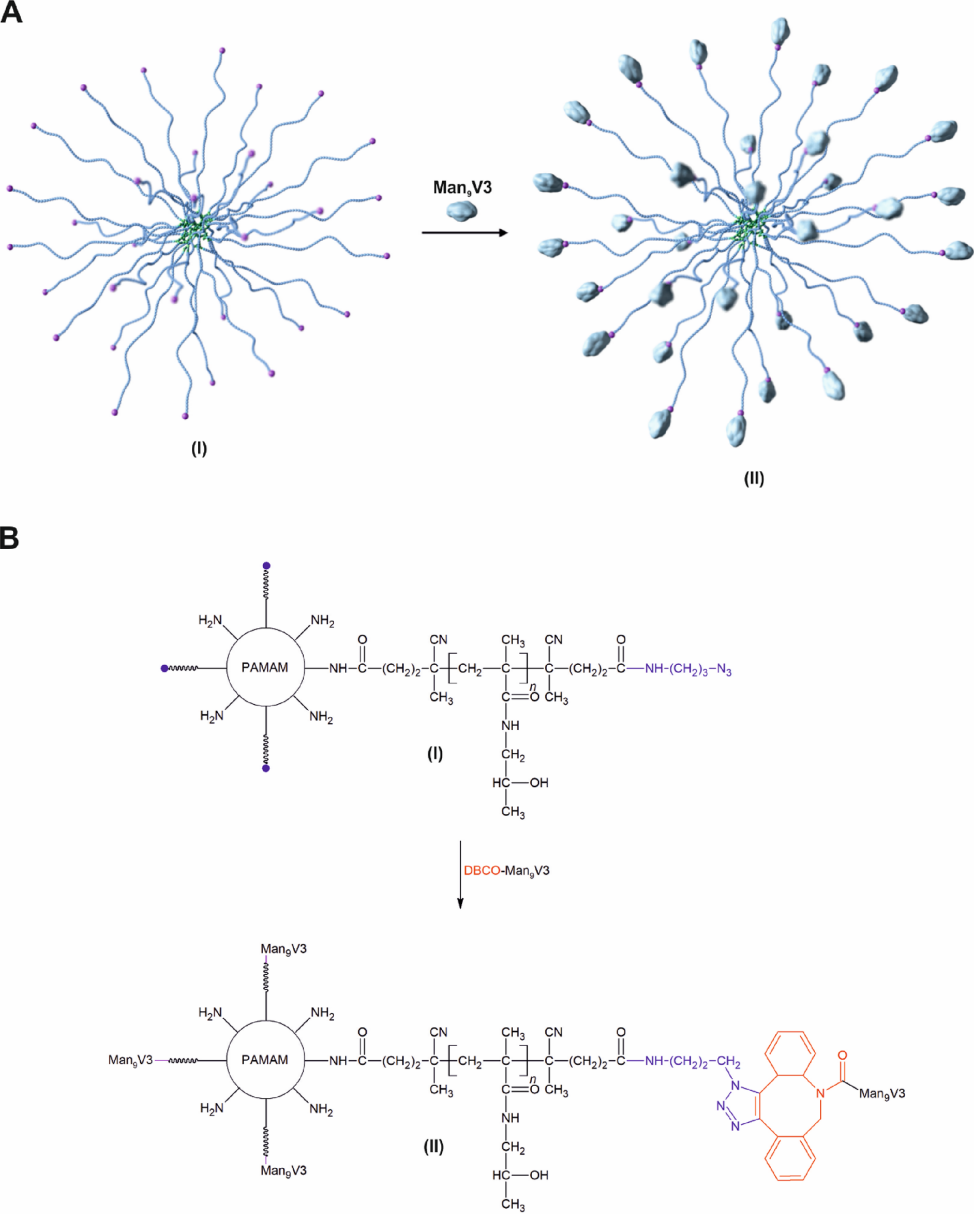
Cartoon Depiction (A) and Reaction Scheme (B) for the Preparation of Star Copolymer Vaccines (II) Synthesized by Conjugation of Man_9_V3 Glycopeptide to Star Copolymers (I)

Star copolymer vaccines **V1**–**V4** were synthesized by reacting purified star copolymers **S27**–**S30** having different lengths and densities of azide-terminated polymer arms with the dibenzocyclooctyne (DBCO)-functionalized V3 glycopeptide (Man_9_V3-DBCO) via a strain-promoted cycloaddition reaction (see Scheme 2). The coupling of (macro)molecules mediated by the interaction between azides and DBCO groups was chosen because of their biorthogonality, fast reaction kinetics, and lack of catalyst and byproducts simplifying purification. The conjugation of Man_9_V3-DBCO to the polymer platform was manifested by an increase in the *M*_n_, from which the number of bound glycopeptide units was calculated. The degree of substitution of the polymer arms by glycopeptide units ranged from ∼66 to 100%, with higher conjugation efficiencies achieved for star copolymers with longer polymer arms. Binding of the glycopeptide also led to an increase in the hydrodynamic radii (*R*_h_), although it should be noted that the radii of gyration (*R*_g_) of the star copolymer vaccines were similar to those of the unmodified star copolymers within experimental measurement error, which is likely due to the lower sensitivity of static light scattering used to determine *R*_g_. Changes in the slope of the Zimm plot, from which *R*_g_ is calculated, are very small for particles <30 nm, while changes in the diffusive motion of the particles, from which *R*_h_ is calculated, are more sensitive to be reliably detected. The *Đ* values of the star copolymer vaccines were very low (*Đ* < 1.3), indicating that conjugation was not accompanied by cross-linking or other undesirable side reactions (see Table 3).

**Table 3:** Characteristics of Star Copolymers **S27**–**S30** (A) and Their Conjugates with the Man_9_V3 Glycopeptide and the Star Copolymer Vaccines **V1**–**V4** (B)

**A**							
	**star copolymer**	**polymer arm**	***M*_n_**^i^ [kg·mol^–1^]	***Đ***^ii^	***R*_g_**iii**[nm]**	***R*_*h*_**^iv^**[nm]**	***N***^v^**(polymer arms)**
	**S27**	P4	275.7	1.11	10.2	12.2	28.5
	**S28**	P5	1890.7	1.16	31.8	33.7	26.1
	**S29**	P4	84.8	1.10	8.7	9.7	9.0
	**S30**	P5	559.1	1.07	21.6	22.9	7.7

**B**							
	**star copolymer vaccine**	**star copolymer**	***M*_n_**^i^ [kg·mol^–1^]	***Đ***^ii^	***R*_g_**iii**[nm]**	*R*_*h*_^iv^**[nm]**	*N*^vi^**(Man**_**9**_**V3 units)**
	**V1**	S27	419.8	1.17	6.2	16.4	18.9
	**V2**	S28	2107.1	1.24	38.4	34.4	28.4
	**V3**	S29	135.9	1.18	n.d.	11.8	6.7
	**V4**	S30	609.7	1.14	18.0	27.0	6.6

i Number-average molecular weight of the star copolymer/star copolymer vaccine determined by SEC.

ii Star copolymer/star copolymer vaccine dispersity defined as the ratio of weight-average (*M*_w_) to number-average (*M*_n_) molecular weight determined by SEC.

iii Radius of gyration of the star copolymer/star copolymer vaccine determined by SEC.

iv Hydrodynamic radius of the star copolymer/star copolymer vaccine determined by DLS.

v Number of polymer arms attached to the PAMAM dendrimer core evaluated by SEC.

vi Number of minimal peptide immunogen units attached to the PHPMA arms of the star copolymer evaluated by SEC.

### Ultrahigh-Resolution Imaging of Star Copolymer Vaccines

**Figure 3:**
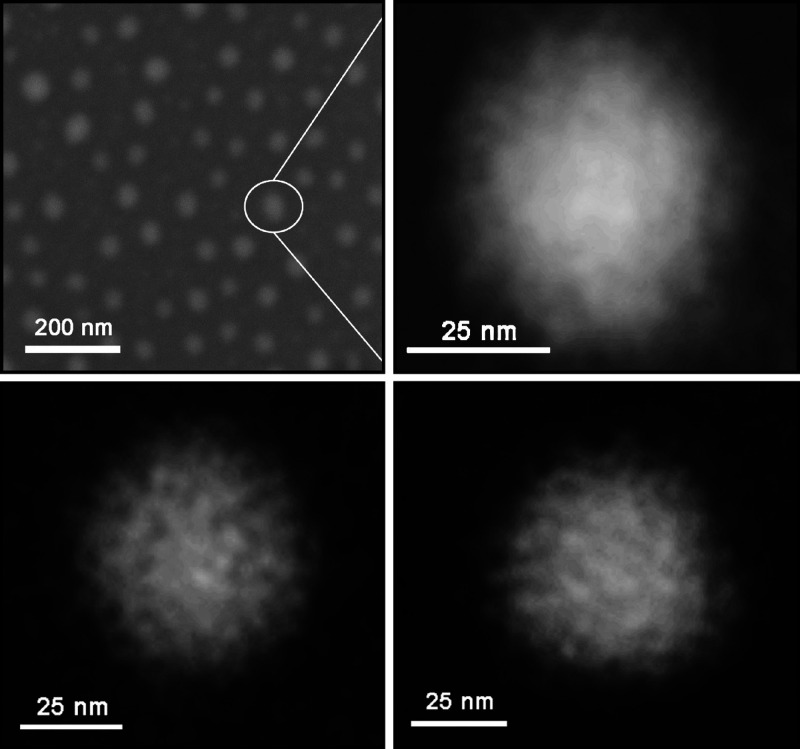
Ultrahigh-resolution micrographs of unlabeled star copolymer vaccine construct with ∼70 kg·mol^–1^ polymer arms obtained by ESEM. The overall image at the top left shows the arrangement of star copolymer vaccine of similar shapes and sizes, while the remaining images show a zoomed-in view of individual ∼50 nm star copolymer vaccines emphasizing their spherical shape and entangled structure of the polymer coils.

To document the size and shape of the star copolymer vaccines not only in solution but also in the dry state, we subjected them to detailed EM analysis. However, while in the case of unmodified star copolymers, it was possible to decorate their surfaces with gold nanoparticles (Au_11_) to enhance the image contrast, this strategy could not be implemented in the case of vaccines because the end groups of the polymer arms were occupied by minimal peptide immunogen molecules. For this reason, the direct visualization of unlabeled star copolymer vaccines by EM was a major challenge. However, as can be seen from the acquired micrographs of a representative star copolymer vaccine with ∼70 kg·mol^–1^ polymer arms (Figure 3), by using a suitable method and optimizing the measurement conditions, it was possible to reliably capture the analyzed subjects. The analyzed vaccine nanoparticles had a spherical shape with a radius of ∼25 nm, which is in close agreement with the values obtained from the DLS measurement.

### Impact of Star Copolymer Arm Density and Molecular Weight on Vaccine Activity

We next assessed whether modular properties of the star copolymer could be tuned to impact immune responses when used as a vaccine delivering the minimal immunogen, Man_9_V3. Star copolymers with two different arm lengths (*M*_n_ target ∼10 or 80 kg·mol^–1^) and number of polymer arms (*N* ∼ 10 or 30) were prepared to generate star copolymer vaccines with varying size and minimal immunogen density as summarized in Table 3 and then assessed for the capacity to induce antibody responses in mice following vaccination.

**Figure 4:**
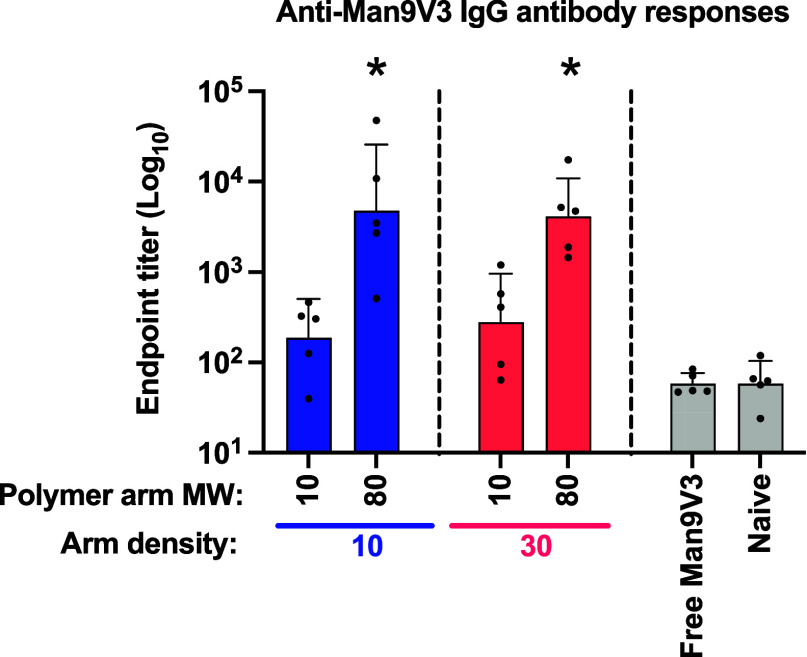
Antibody responses following vaccination with different star copolymer vaccine compositions, free (unformulated Man_9_V3) or naïve control. Balb/c mice (*n* = 5/group) were immunized at days 0, 28, and 56 and serum was collected on day 70 and assessed for anti-Man_9_V3 IgG antibodies by enzyme-linked immunosorbent assay (ELISA). Data are presented as the mean ± standard deviation end point titer. Differences between each experimental group and the naïve control were assessed for statistical significance using one-way ANOVA with Bonferroni correction for multiple comparisons; asterisks (*) indicate statistical significance (*P* < 0.05) between the indicated group and naïve.

Whereas vaccination with the soluble monomeric Man_9_V3 (“Free Man_9_V3”) did not induce antibodies above background levels in naïve, untreated animals, all the star copolymer compositions induced detectable antibody responses (Figure 4). Furthermore, there was a clear trend between increasing arm *M*_n_ and increased antibody responses with the star copolymers with 80 kg·mol^–1^ arms (**V2** and **V4**) inducing antibody titers that were greater than 10-fold higher than the star copolymers with 10 kg·mol^–1^ arms (**V1** and **V3**) and nearly 100-fold and statistically significantly higher than background levels observed in naïve animals. No differences in antibody responses were observed with star copolymers having 10 or 30 arms, which suggests that the star copolymer size may be more critical than the polymer arm density (number of polymer arms) in promoting antibody responses. Of note, the responses observed at 2 weeks after the final vaccination (day 70), corresponding to the peak of the antibody response, are comparable to the levels observed against Man_9_V3 in other studies that were considered biologically meaningful.^33,36^

While these preliminary data highlight some potential advantages of the star copolymer as a tunable platform for delivering minimal immunogens to modulate antibody responses, further immunological assessment characterizing how such parameters impact pharmacokinetics and biodistribution as well as uptake and processing by innate immune cell populations to influence antibody and T cell responses will be needed. For instance, prior studies have established that increasing the hydrodynamic size of particulate vaccines can lead to greater uptake by immune cell subsets in lymph nodes associated with enhanced antibody and T cell responses.^13,37−39^ Therefore, future studies assessing how star copolymer arm length (and hydrodynamic size) impacts distribution to and uptake by immune cells in lymph nodes may be helpful for understanding the mechanistic basis for the results presented herein.

### Star Copolymer Vaccines Have Excellent Recovery Following Sterile Filtration

Sterile filtration to ensure product sterility is a key step in vaccine manufacturing. A potential advantage of star copolymers is that their size can be tuned and tightly controlled between about 10 and 50 nm diameter, which is well below most sterile filter pore sizes of about 0.2 μm. To assess the capacity of the star copolymer vaccines to undergo sterile filtration, a representative star-Man_9_V3 vaccine with 30 fully conjugated ∼10 kg·mol^–1^ polymer arms was filtered through 0.2 μm pore filters comprising either polytetrafluoroethylene (PTFE), nylon, polyethlysulfone (PES), or cellulose acetate (CA) and then assessed for material properties and recovery by SEC and high-performance liquid chromatography (HPLC). Notably, the star copolymer vaccine showed excellent recovery with no impact on particle size and molecular weight following filtration from PTFE, nylon, and PES, whereas no star copolymer vaccine was recoverable following filtration through CA (see Table S2). These data show that star copolymer vaccines can be efficiently and nondestructively filtered through conventional filtration membranes (except CA), thereby ensuring sterility required for injectables intended for human use.

### Stability of Star Copolymers and Star Copolymer Vaccines in Aqueous Buffer

For commercial utility, star copolymer vaccines should have sufficient stability in aqueous buffers during manufacturing, storage, and solutions prior to administration in the clinical or hospital setting. First, we assessed the stability of a representative star copolymer with ∼10 PHPMA arms of *M*_n_ ∼ 30 kg·mol^–1^ placed at 37 °C in a temperature-controlled environment and then sampled at up to 16 weeks to assess for degradation by DLS and SEC. Notably, the star copolymer showed excellent stability at 37 °C; 10% was degraded through 2 weeks, which increased to 15% degraded by 4 weeks and more than 40% degraded by 16 weeks (Table S3A and Figure S4). The gradual degradation of the star copolymer was probably due to the retro-Michael reaction occurring in the PAMAM dendrimers, which is especially favored in aqueous solutions at elevated temperatures. As a consequence, a branching methacrylamide chain (conjugated with PHPMA arm) is cleaved, and a defective dendrimer with one missing branch is formed.^40^ We further evaluated the stability of the representative star copolymer vaccine comprising Man_9_V3 glycopeptide (∼10 units) stored under various conditions, including 25 °C for 5 days and 4 °C and −20 °C for up to 16 weeks either as a lyophilized solid or in PBS solution. The results of the DLS and SEC analyses showed that the values of all monitored quantities (*R*_h_, *M*_w_, and *Đ*) remained within the experimental error practically unchanged (Table S3B and Figure S5). Overall, these data clearly demonstrate that the star copolymers based on PAMAM and PHPMA as well as their conjugates with Man_9_V3 have excellent stability under manufacturing, handling, and storage conditions, which bodes well for their potential use in commercial applications.

## Conclusions

The presented article discusses the synthesis and optimization of the structure of star copolymers as tunable nanoparticle carriers of minimal immunogens for use as vaccines. Specifically, we studied how the length of PHPMA arms, dendrimer generation, and molar ratio of arms to dendrimer functional groups impact star copolymer size, arm density, and yield. In addition, ultrahigh-resolution images of a selected star copolymer were provided to document its size and shape in the unsolvated state. Three important conclusions can be drawn from the measured data: (i) the polymer arms with a higher *M*_n_ form larger-sized star copolymers, but they attach to the surface of the dendrimer to a lower extent than the polymer arms with lower *M*_n_; (ii) the branching degree (generation) of the dendrimer can control the density of the arms extending from the dendrimer core, but not the size of the star copolymer; and (iii) the use of a higher molar ratio of PAMAM **~**NH_2_ groups to PHPMA **~**TT groups leads to a lower polymer arm density but higher star copolymer yield, regardless of the polymer arm length and PAMAM generation used. Finally, with an eye to clinical translation, we investigated how the modular parameters of star copolymers impact their activity for use as minimal immunogen vaccines using a glycopeptide derived from the envelope protein of the HIV-1 virus as a representative minimal immunogen. The results showed that all star copolymer vaccine compositions elicited a detectable antibody response, with the effect being more pronounced for the larger star copolymers with longer arms, while the number of polymer arms had little effect on the antibody titer. Considering the long-term storage stability as refrigerated (4 °C) or frozen (−20 °C) solutions or even better as lyophilized solids and excellent yields after sterile filtration, star copolymer vaccines represent a very promising platform for minimal immunogen vaccines.

## Experimental Procedures

### Chemicals

Acetic anhydride, (*RS*)-1-aminopropan-2-ol, l-ascorbic acid, 3-azidopropylamine, 4,4′-azobis(4-cyanovaleric acid) (ACVA), copper(I) bromide (CuBr), copper(II) sulfate pentahydrate (CuSO_4_·5H_2_O), 4-cyano-4-(phenylcarbonothioylthio)pentanoic acid (CPP), *N*,*N*′-dicyclohexylcarbodiimide (DCC), *N*-(3-(dimethylamino)propyl)-*N′*-ethylcarbodiimide hydrochloride (EDC), 4-(dimethylamino)pyridine (DMAP), 8-hydroxyquinoline, methacryloyl chloride, sodium carbonate, thiazolidine-2-thione (TT), and tris[(1-benzyl-1H-1,2,3-triazol-4-yl)methyl]amine (TBTA) were purchased from Sigma-Aldrich, Czech Republic and used as received. Polyamidoamine dendrimers, ethylendiamine core, generation 3.0–5.0 (PAMAM, G3–5) were purchased from Sigma-Aldrich, Czech Republic. Mono-Sulfo-NHS-Undecagold (Au_11_) was obtained from Nanoprobes, NY, USA. The Man_9_V3-DBCO glycopeptide was kindly provided by Chemitope Glycopeptide, NY, USA. All compounds are >95% pure by HPLC analysis. All solvents were of HPLC grade and dried over a layer of activated molecular sieves (4 Åm) before use.

### Synthesis of Monomer, Functionalized Chain Transfer Agent, and Initiators

*N*-(2-Hydroxypropyl)methacrylamide (HPMA) was synthesized by reacting methacryloyl chloride with (RS)-1-aminopropan-2-ol in dichloromethane in the presence of sodium carbonate as described in reference.^41^

Dithiobenzoic acid 1-cyano-1-methyl-4-oxo-4-(2-thioxothiazolidin-3-yl)butyl ester (CPP-TT) was prepared by the reaction of CPP with TT in dichloromethane in the presence of DCC and DMAP.^42^

Dithiobenzoic acid 3-(3-azidopropylcarbamoyl)-1-cyano-1-methylpropyl ester (CPP-N_3_) was produced by the reaction of CPP with 3-azidopropylamine in ethyl acetate in the presence of EDC.^43^

2-[1-Cyano-1-methyl-4-oxo-4-(2-thioxo-thiazolidin-3-yl)-butylazo]-2-methyl-5-oxo-5-(2-thioxothiazolidin-3-yl)-pentanenitrile (ACVA-(TT)_2_) was prepared by the reaction of ACVA with TT in tetrahydrofuran in the presence of DCC and DMAP.^44^

4-Cyano-4-(1-cyano-3-ethynylcarbamoyl-1-methylpropylazo)-*N*-ethynyl-4-methylbutyramide (ACVA-(Pg)_2_) was synthesized by reacting ACVA with propargylamine in dichloromethane in the presence of EDC and DMAP.^43^

*N*-(3-Azidopropyl)-4-[3-(3-azidopropylcarbamoyl)-1-cyano-1-methylpropylazo]-4-cyano-4-methylbutyramide (ACVA-(N_3_)_2_) was synthesized by reacting ACVA with 3-azidopropylamine in dichloromethane in the presence of EDC and DMAP.^45^

### Synthesis of Functionalized Immunostimulant and Minimal Peptide Immunogen

1-(4-(Aminomethyl)benzyl)-2-butyl-1*H*-imidazo[4,5-c]quinolin-4-amine (2Bxy) was prepared by a multistep reaction starting from quinoline-2,4-diol as previously described.^11^

Man_9_V3, a glycosylated HIV V3 minimal peptide immunogen of the composition EINCTRPNNNTRPGEIIGDIRQAHCNISRA with a C-terminal PEG_3_-DBCO linker, was synthesized following a procedure similar to that described in ref (21), except that the C-terminus of the glycopeptide was linked to a Boc-amine-PEG_3_ linker instead of biotin as shown in Scheme S1. Briefly, Man_9_GlcNAc_2_–NH_2_ (5) was originally synthesized using complex glycosyl coupling chemistry while *N*-terminal fragment (4) and Boc-amine-PEG_3_-linked *C*-terminal fragment (1) were acquired through solid-phase peptide synthesis. Man_9_GlcNAc_2_ glycosyl amine (5) was attached to the free carboxylic acid side chain at position 301 on the *N*-terminal fragment (4) and at position 332 on the Boc-amine-PEG_3_ linked *C*-terminal fragment (1) to provide glycopeptide thioester (7) and *N*-terminal cysteinyl glycopeptide (8), respectively. These two fragments were then coupled using native chemical ligation, immediately followed by cyclization via disulfide formation to yield the Man_9_V3-PEG_3_-amine. DBCO-NHS ester was then introduced to react with the amine group, affording the final product Man_9_V3-DBCO (11).

### Synthesis of Heterobifunctional Polymer Arms

Polymer arms **P1–P3** were synthesized by RAFT polymerization of HPMA in a *tert*-butyl alcohol/DMSO mixture in the presence of various amounts of CPP-TT and ACVA-(TT)_2_ (see Table 1). Polymer arms **P4** and **P5** were prepared under the same conditions but in the presence of CPP-N_3_ and ACVA-(N_3_)_2_. In the second step, the dithiobenzoate (DTB) end groups of the polymers were capped by the homolytic reaction with an excess of functionalized initiators, ACVA-(Pg)_2_ in the case of polymer arms **P1–P3**, and ACVA-(TT)_2_ in the case of polymer arms **P4** and **P5**, to form propargyl (Pg) or carbonylthiazolidine-2-thione (TT) groups, respectively. Below is given a typical procedure for the synthesis of heterobifunctional polymer **P1**.

A mixture of CPP-TT (39.4 mg, 103.0 μmol) and ACVA-(TT)_2_ (24.9 mg, 51.5 μmol) was dissolved in 1.6 mL of DMSO and added to a solution of HPMA (2.0 g, 14.0 mmol) in 14.1 mL of *tert*-butanol. The reaction mixture was thoroughly bubbled with argon and polymerized in a sealed glass ampule at 70 °C for 16 h. Then, the mixture was precipitated into 300 mL of acetone/diethyl ether (3:1), and the precipitate formed was filtered, redissolved in methanol, and precipitated again into the same precipitant. After drying under vacuum, 828 mg (41%) of the product was obtained as an orange, amorphous powder. The number-average molecular weight (*M*_n_) and the dispersity (*Đ*) of the polymer precursor were 9.2 kg·mol^–1^ and 1.03, respectively.

A mixture of polymer precursor (200.0 mg, 21.7 μmol of DTB gr.) and ACVA-(Pg)_2_ (231.1 mg, 652.2 μmol) was dissolved in DMSO (2 mL), thoroughly bubbled with argon, and incubated at 80 °C for 3 h. Then, the mixture was precipitated into 40 mL of acetone/diethyl ether (3:1), the precipitate formed was filtered, redissolved in methanol, and precipitated again into the same precipitant. After drying under vacuum, 154 mg (77%) of the **P1** polymer was obtained as a pale-yellow amorphous powder. The *M*_n_ and *Đ* of the **P1** polymer were 9.8 kg·mol^–1^ and 1.03, respectively. The molar content of the thiazolidine-2-thione (TT) end group of the polymer was 98.9 μmol/g, corresponding to an average functionality of 0.97.

### Synthesis of Star Copolymers

Star copolymers were synthesized by acylation of the PAMAM dendrimer of various generations (G3, G4, and G5) with a heterobifunctional polymer arm of different molecular weights (10, 16, 41, and 96 kDa) at different molar ratios of the PAMAM amino groups to the TT terminal group on the polymer arms (1:1, 2:1, and 3:1). Below is given a typical procedure for the synthesis of star copolymer **S1**.

Methanolic solution of PAMAM, G3 (1.1 μL of 20 wt %, 30.3 nmol of ~NH_2_ gr.) was added to a solution of linear polymer **P1** (9.8 mg, 0.97 μmol of TT gr.) in 100 μL of methanol, and the reaction mixture was shaken at 25 °C for 16 h. Then, the mixture was precipitated into 2.0 mL of diethyl ether; the precipitate formed was collected by centrifugation, redissolved in water, and freeze-dried to give 9.5 mg of a mixture of star copolymer **S1** and linear polymer **P1**. The *M*_n_, *Đ*, radius of gyration (*R*_g_) and weight fraction of star copolymer **S1** in the isolated product were 246.6 kg·mol^–1^, 1.12, 9.6 nm, and 48.7%, respectively.

A selected star copolymer **S17** was further purified from the unreacted linear polymers by membrane filtration using RC centrifugal filter units with molecular weight cutoff (MWCO) 100 kg·mol^–1^ (Sigma-Aldrich, Czech Republic) in PBS (4×) and in H_2_O (2×) and isolated by lyophilization. For SEC chromatograms of star copolymer **S17** before and after the purification, see Figure S1.

### Labeling of Star Copolymers with the Contrast Agent

The purified star copolymer **S17** was labeled by reaction with an Au-based contrast agent (Au_11_). The Au_11_ nanoparticles were covalently linked to either (i) the surface of the PAMAM core, (ii) the end groups of the PHPMA arms, or (iii) both the PAMAM core and the ends of the PHPMA arms. In addition, in the case of approach (i), different numbers (30, 15, and 5) of Au_11_ nanoparticles were attached to the ends of the polymer arms. An example of the preparation of a star copolymer labeled with approximately 5 Au_11_ nanoparticles attached to the ends of PHPMA arms is described below:

The purified star copolymer **S17** (171.8 mg, 47 μmol of −NH_2_ gr.) was dissolved in 3.436 mL of *N,N’*-dimethylacetamide (DMAc) and mixed with 45 μL of acetic anhydride (470 μmol), and the solution was shaken at 25 °C for 2 h. The reaction mixture was diluted with methanol (1:1) and separated on a column filled with Sephadex LH-20 (Sigma-Aldrich, Czech Republic) in methanol. The polymer fraction was precipitated into diethyl ether yielding 165 mg of the star copolymer with acetylated amino groups on the surface of PAMAM.

Afterward, the copolymer (155.0 mg, 11 μmol of Pg gr.) was dissolved in 3.2 mL of DMAc, 3-azidopropylamine (2.2 μL, 22 μmol), TBTA (5.9 mg, 11 μmol), and CuBr (1.6 mg, 11 μmol) were added, and the reaction mixture was thoroughly bubbled with Ar. After 24 h, the reaction mixture was diluted by the addition of 8-hydroxyquinoline and separated on a Sephadex LH-20 column in methanol. The polymer fraction was precipitated into diethyl ether, yielding 147 mg of the star copolymer with the primary amino groups at the ends of the PHPMA arms.

Finally, an aqueous solution of the star copolymer (0.64 mg, 46 nmol of ~NH_2_ gr.) was mixed with the Au_11_ nanoparticles (8 nmol) dissolved in 1 mL of 10% isopropanol in water and the reaction mixture was shaken overnight at 25 °C. The resulting product was purified on a PD-10 column (Sigma-Aldrich, Czech Republic) in H_2_O and lyophilized to give 0.58 mg of the star copolymer with approximately 5 Au_11_ molecules attached to the ends of the PHPMA arms.

### Synthesis of Star Copolymer Vaccines

Star copolymer vaccines comprising a G5 PAMAM dendrimer core with different lengths and densities of PHPMA polymer arms were prepared as described above, except the polymer arms (**P4** and **P5**) were terminated with an azide group used to link the HIV minimal peptide immunogen, Man_9_V3 bearing a dibenzylcyclooctyl (DBCO) group, to the star copolymer via strain-promoted azide–alkyne cycloaddition. For example, star copolymer vaccine **V3** was prepared by mixing 1.09 mg of star copolymer **S29** (0.116 μmol of ~N_3_ group) dissolved in 1.1 μL of DMSO/DMF (1/1 v/v) and 0.91 mg of Man_9_V3-DBCO (0.119 μmol) in 18.3 μL of DMSO/DMF (1/1 v/v). The reaction of star copolymer with a slight, 3 mol % excess of Man_9_V3 (or 1:1.03 molar ratio) was allowed to proceed at 25 °C overnight. An aliquot was then injected onto the HPLC instrument, and conversion was evaluated by comparing the areas under the curve of unreacted Man_9_V3-DBCO before and after the reaction. If free Man_9_V3-DBCO (>3%) was present in the reaction mixture, additional star copolymer was added to consume the remaining glycopeptide until its content was below 3%. The star copolymer vaccine was lyophilized, redissolved in 1× PBS, and stored at −20 °C before use for animal studies. The *M*_n_ and *Đ* of star copolymer vaccine **V3** were 135.9 kg·mol^–1^ and 1.18, respectively.

### Size-Exclusion Chromatography

The number- and weight-average molecular weights (*M*_n_ and *M*_w_), dispersities (*Đ*), and gyration radii (*R*_g_) of all linear polymer arms as well as the star copolymers were determined by size-exclusion chromatography (SEC) on an HPLC system (Shimadzu Corp., Japan) equipped with internal UV–VIS diode array detector (SPD-M20A) and external differential refractometer (Optilab T-rEX) and multiangle light scattering detector (DAWN HELEOS II, both Wyatt Technology Corp., CA, USA). TSKgel SuperAW3000 and SuperAW4000 columns (Tosoh Bioscience, PA, USA) in series were used to analyze samples in a mobile phase of 80% methanol/20% sodium acetate buffer (0.3 M, pH 6.5) at a flow rate of 0.6 mL·min^–1^. The d*n*/d*c* values of 0.168 and 0.176 mL·g^–1^ were used to calculate the molecular weights of linear/star copolymers and star copolymer vaccines, respectively.

### High-Performance Liquid Chromatography

The purity of low-molecular-weight compounds, including monomer, chain transfer agents, initiators, and immunostimulant, was verified on a high-performance liquid chromatography (HPLC) system (Shimadzu, Japan) equipped with an internal UV–vis diode array (SPD-M20A) and ELSD (LTII) detectors using the Chromolith HighResolution RP-18e reverse-phase column (Merck, USA), with a linear gradient (0–100%) of water–acetonitrile mixture containing 0.1% TFA at a flow rate of 2.5 mL·min^–1^. The course of conjugation of Man_9_V3-DBCO glycopeptide to the star copolymers was monitored by an HPLC system (Agilent 1260 Infinity II, CA, USA) equipped with an internal UV–vis diode array detector using an Agilent InfinityLab Poroshell 120 EC-C18 reverse-phase column, with a linear gradient (5–65%) of water–acetonitrile mixture containing 0.05% TFA at a flow rate of 1.25 mL·min^–1^.

### UV–vis Spectrophotometry

The spectrophotometric analyses of functionalized linear polymers were performed in quartz glass cuvettes on a Specord Plus UV–vis spectrophotometer (Analytik Jena, Jena, Germany). The molar content of the terminal DTB and TT groups in the polymers was determined at 302 and 305 nm in methanol using the molar absorption coefficient of 12,100 and 10,300 L·mol^–1^·cm^–1^, respectively.

### Dynamic Light Scattering

The hydrodynamic sizes of purified star copolymers were determined by the dynamic light scattering (DLS) technique at a scattering angle of 173° using a Nano-ZS instrument (Malvern Instruments, UK) equipped with a 4 mW, 633 nm laser. Measurements were performed at a sample concentration of 1.0 mg·mL^–1^ in PBS buffers (0.15 M, pH 7.4) at 37 °C. For the evaluation of the dynamic light scattering data, the DTS (Nano) program was used. The resulting hydrodynamic sizes were arithmetic means of at least 10 independent measurements.

### Electron Microscopy

Ultrahigh-resolution electron microscopy imaging of Au-labeled star copolymers and unlabeled star copolymer vaccines was performed on a custom-modified environmental scanning electron microscope (ESEM) Quanta 650 FEG (Thermo Fisher Scientific, MA, USA)^46^ equipped with a detector for scanning transmission electron microscopy (STEM). The lyophilized samples were dissolved in distilled water and applied to a lacey carbon film on a copper grid. The solution of star copolymers in water (2 μL) was applied on a TEM grid covered with holey carbon film.^47,48^ Then, the samples were *in situ* freeze-dried at 20 °C and 10 Pa in the ESEM specimen chamber^49^ (operated under environmental mode). Observation was performed at a beam energy of 30 keV, beam current of 5 pA, and working distance of 5.3 mm in high vacuum mode using a dark-field STEM detector. Micrographs were postprocessed using MountainsSEM software (Digital Surf, France).

### *In Vivo* Vaccination

Animals were housed and cared for in accordance with the American Association for Accreditation of Laboratory Animal Care standards in accredited facilities at the Vaccine Research Center, and all animal procedures were performed according to a protocol approved by the Institutional Animal Care and Use Committees of the National Institute of Allergy and Infectious Diseases, National Institutes of Health.

Female Balb/c mice, 8–12 weeks of age, were obtained from The Jackson Laboratory (Bar Harbor, ME, USA) and maintained at the Vaccine Research Center’s (VRC) Animal Care Facility (Bethesda, MD, USA) under pathogen-free conditions. Star copolymers for vaccination were prepared as 25 μg of Man_9_V3 minimal peptide immunogen equivalent star copolymer in PBS buffer with adjuvant comprising 5 μg PADRE and 5 nmol of the TLR-7/8 agonist, 2BXy, as previously described.^33^ Immunizations were given intramuscularly at days 0, 28, and 56 and blood was sampled at day 70 to isolate serum for assessment of antibody responses.

### Antibody Measurements

Pierce Streptavidin Coated Plates (Thermo Scientific) were coated overnight at 4 °C with a Man_9_V3-biotin probe in PBS buffer. Plates were then blocked with PBS + FCS, and serum was applied in serial 10-fold dilutions and incubated at 37 °C. Detection was performed at room temperature with total antimouse IgG HRP-conjugated secondary antibodies (1:6000, Southern Biotech) followed by TMB + substrate–chromogen (Dako) and a 2N sulfuric acid stop solution. Washing was performed between steps with PBS + 0.05% Tween 20. Plates were read on a spectrophotometer (and data were analyzed in Prism (GraphPad). End point titers were determined by fitting data using a four-parameter dose–response curve.

## Data Availability

The data underlying this study are available in the published article and its Supporting Information.

## Supporting Information Available

Reaction scheme for the preparation of Man_9_V3-DBCO (Scheme S1), characteristics of PAMAM dendrimers (Table S1), SEC chromatograms of unpurified and purified star copolymer S17 (Figure S1), SEC chromatograms of heterobifunctional polymer arms P1–P3 (Figure S2), *R*_h_-distribution function of purified star copolymer S17 (Figure S3), size parameters and mass recoveries of star copolymer vaccine solutions before and after filtration (Table S2), size parameters of star copolymers and star copolymer vaccines stored under different conditions (Table S3), SEC chromatograms of star copolymers stored under different conditions (Figure S4), and SEC chromatograms of star copolymer vaccines stored under different conditions (Figure S5) (PDF)

## References

[ref1] Ross T. M. Universal Influenza Vaccine Approaches Using Full-Length or Head-Only Hemagglutinin Proteins. J. Infect Dis 2019, 219, S57–S61. 10.1093/infdis/jiz004. 30715379

[ref2] Li Y.-D.; Chi W.-Y.; Su J.-H.; Ferrall L.; Hung C.-F.; Wu T.-C. Coronavirus vaccine development: from SARS and MERS to COVID-19. J. Biomed. Sci. 2020, 27, 104 10.1186/s12929-020-00695-2. 33341119 PMC7749790

[ref3] Plotkin S. A. Correlates of Protection Induced by Vaccination. Clin Vaccine Immunol 2010, 17, 1055–1065. 10.1128/CVI.00131-10. 20463105 PMC2897268

[ref4] de Taeye S. W.; de la Peña A. T.; Vecchione A.; Scutigliani E.; Sliepen K.; Burger J. A.; van der Woude P.; Schorcht A.; Schermer E. E.; van Gils M. J.; et al. Stabilization of the gp120 V3 loop through hydrophobic interactions reduces the immunodominant V3-directed non-neutralizing response to HIV-1 envelope trimers. J. Biol. Chem. 2018, 293, 1688–1701. 10.1074/jbc.RA117.000709. 29222332 PMC5798299

[ref5] Arvin A. M.; Fink K.; Schmid M. A.; Cathcart A.; Spreafico R.; Havenar-Daughton C.; Lanzavecchia A.; Corti D.; Virgin H. W. A perspective on potential antibody-dependent enhancement of SARS-CoV-2. Nature 2020, 584, 353–363. 10.1038/s41586-020-2538-8. 32659783

[ref6] Kozak M.; Hu J. The Integrated Consideration of Vaccine Platforms, Adjuvants, and Delivery Routes for Successful Vaccine Development. Vaccines 2023, 11, 695 10.3390/vaccines11030695. 36992279 PMC10055765

[ref7] Pollard A. J.; Bijker E. M. A guide to vaccinology: from basic principles to new developments. Nat. Rev. Immunol 2021, 21, 83–100. 10.1038/s41577-020-00479-7. 33353987 PMC7754704

[ref8] Irvine D. J.; Read B. J. Shaping humoral immunity to vaccines through antigen-displaying nanoparticles. Curr. Opin Immunol 2020, 65, 1–6. 10.1016/j.coi.2020.01.007. 32200132 PMC7501207

[ref9] Purcell A. W.; McCluskey J.; Rossjohn J. More than one reason to rethink the use of peptides in vaccine design. Nat. Rev. Drug Discovery 2007, 6, 404–414. 10.1038/nrd2224. 17473845

[ref10] Malonis R. J.; Lai J. R.; Vergnolle O. Peptide-Based Vaccines: Current Progress and Future Challenges. Chem. Rev. 2020, 120, 3210–3229. 10.1021/acs.chemrev.9b00472. 31804810 PMC7094793

[ref11] Lynn G. M.; Laga R.; Darrah P. A.; Ishizuka A. S.; Balaci A. J.; Dulcey A. E.; Pechar M.; Pola R.; Gerner M. Y.; Yamamoto A.; et al. In vivo characterization of the physicochemical properties of polymer-linked TLR agonists that enhance vaccine immunogenicity. Nat. Biotechnol. 2015, 33, 1201 10.1038/nbt.3371. 26501954 PMC5842712

[ref12] Dintzis H. M.; Dintzis R. Z.; Vogelstein B. Molecular determinants of immunogenicity: the immunon model of immune response. Proc. Natl. Acad. Sci. U. S. A. 1976, 73, 3671–5. 10.1073/pnas.73.10.3671. 62364 PMC431180

[ref13] Bachmann M. F.; Jennings G. T. Vaccine delivery: a matter of size, geometry, kinetics and molecular patterns. Nature Reviews Immunology 2010, 10, 787–796. 10.1038/nri2868. 20948547

[ref14] Brito L. A.; O’Hagan D. T. Designing and building the next generation of improved vaccine adjuvants. J. Controlled Release 2014, 190, 563–579. 10.1016/j.jconrel.2014.06.027. 24998942

[ref15] Irvine D. J.; Hanson M. C.; Rakhra K.; Tokatlian T. Synthetic Nanoparticles for Vaccines and Immunotherapy. Chem. Rev. 2015, 115, 11109–11146. 10.1021/acs.chemrev.5b00109. 26154342 PMC4688911

[ref16] Morales-Hernández S.; Ugidos-Damboriena N.; López-Sagaseta J. Self-Assembling Protein Nanoparticles in the Design of Vaccines: 2022 Update. Vaccines 2022, 10, 1447 10.3390/vaccines10091447. 36146525 PMC9505534

[ref17] Lamontagne F.; Khatri V.; St-Louis P.; Bourgault S.; Archambault D. Vaccination Strategies Based on Bacterial Self-Assembling Proteins as Antigen Delivery Nanoscaffolds. Vaccines 2022, 10, 1920 10.3390/vaccines10111920. 36423016 PMC9696568

[ref18] Montégut L.; Chen H.; Bravo-San Pedro J. M.; Motiño O.; Martins I.; Kroemer G. Immunization of mice with the self-peptide ACBP coupled to keyhole limpet hemocyanin. Star Protoc 2022, 3, 101095 10.1016/j.xpro.2021.101095. 35059656 PMC8760546

[ref19] Doucet M.; El-Turabi A.; Zabel F.; Hunn B. H. M.; Bengoa-Vergniory N.; Cioroch M.; Ramm M.; Smith A. M.; Gomes A. C.; Cabral de Miranda G.; Wade-Martins R.; Bachmann M. F.; Kahle P. J.; et al. Preclinical development of a vaccine against oligomeric alpha-synuclein based on virus-like particles. PLoS One 2017, 12, e0181844 10.1371/journal.pone.0181844. 28797124 PMC5552317

[ref20] Kanekiyo M.; Wei C. J.; Yassine H. M.; McTamney P. M.; Boyington J. C.; Whittle J. R. R.; Rao S. S.; Kong W. P.; Wang L. S.; Nabel G. J. Self-assembling influenza nanoparticle vaccines elicit broadly neutralizing H1N1 antibodies. Nature 2013, 499, 102 10.1038/nature12202. 23698367 PMC8312026

[ref21] Alam S. M.; Aussedat B.; Vohra Y.; Meyerhoff R. R.; Cale E. M.; Walkowicz W. E.; Radakovich N. A.; Anasti K.; Armand L.; Parks R.; Sutherland L.; Scearce R.; Joyce M. G.; Pancera M.; Druz A.; Georgiev I. S.; Von Holle T.; Eaton A.; Fox C.; Reed S. G.; Louder M.; Bailer R. T.; Morris L.; Abdool-Karim S. S.; Cohen M.; Liao H. X.; Montefiori D. C.; Park P. K.; Fernández-Tejada A.; Wiehe K.; Santra S.; Kepler T. B.; Saunders K. O.; Sodroski J.; Kwong P. D.; Mascola J. R.; Bonsignori M.; Moody M. A.; Danishefsky S.; Haynes B. F.; et al. Mimicry of an HIV broadly neutralizing antibody epitope with a synthetic glycopeptide. Sci. Transl Med. 2017, 9, eaai7521 10.1126/scitranslmed.aai7521. 28298421 PMC5562351

[ref22] Jefferis R. Posttranslational Modifications and the Immunogenicity of Biotherapeutics. J. Immunol. Res. 2016, 2016, 5358272 10.1155/2016/5358272. 27191002 PMC4848426

[ref23] Li W.; Li F.; Zhang X.; Lin H.-K.; Xu C. Insights into the post-translational modification and its emerging role in shaping the tumor microenvironment. Signal Transduction Targeted Ther. 2021, 6, 422 10.1038/s41392-021-00825-8. PMC8685280 34924561

[ref24] Viegas C.; Seck F.; Fonte P. An insight on lipid nanoparticles for therapeutic proteins delivery. J. Drug Delivery Sci. Technol. 2022, 77, 103839 10.1016/j.jddst.2022.103839.

[ref25] Gouveia M. G.; Wesseler J. P.; Ramaekers J.; Weder C.; Scholten P. B. V.; Bruns N. Polymersome-based protein drug delivery – quo vadis?. Chem. Soc. Rev. 2023, 52, 728–778. 10.1039/D2CS00106C. 36537575 PMC9890519

[ref26] Scaletti F.; Hardie J.; Lee Y. W.; Luther D. C.; Ray M.; Rotello V. M. Protein delivery into cells using inorganic nanoparticle-protein supramolecular assemblies. Chem. Soc. Rev. 2018, 47, 3421–3432. 10.1039/C8CS00008E. 29537040 PMC5962404

[ref27] Skoulas D.; Fattah S.; Wang D.; Cryan S.; Heise A. Systematic Study of Enzymatic Degradation and Plasmid DNA Complexation of Mucus Penetrating Star-Shaped Lysine/Sarcosine Polypept(o)ides with Different Block Arrangements. Macromol. Biosci. 2022, 22, 2200175 10.1002/mabi.202200175. 35634688

[ref28] England R. M.; Moss J. I.; Gunnarsson A.; Parker J. S.; Ashford M. B. Synthesis and Characterization of Dendrimer-Based Polysarcosine Star Polymers: Well-Defined, Versatile Platforms Designed for Drug-Delivery Applications. Biomacromolecules 2020, 21, 3332–3341. 10.1021/acs.biomac.0c00768. 32672451

[ref29] Mehta D.; Leong N.; McLeod V. M.; Kelly B. D.; Pathak R.; Owen D. J.; Porter C. J. H.; Kaminskas L. M. Reducing Dendrimer Generation and PEG Chain Length Increases Drug Release and Promotes Anticancer Activity of PEGylated Polylysine Dendrimers Conjugated with Doxorubicin via a Cathepsin-Cleavable Peptide Linker. Mol. Pharmaceut 2018, 15, 4568–4576. 10.1021/acs.molpharmaceut.8b00581. 30107748

[ref30] Chavoustie S. E.; Carter B. A.; Waldbaum A. S.; Donders G. G. G.; Peters K. H.; Schwebke J. R.; Paull J. R. A.; Price C. F.; Castellarnau A.; McCloud P.; et al. Two phase 3, double-blind, placebo-controlled studies of the efficacy and safety of Astodrimer 1% Gel for the treatment of bacterial vaginosis. Eur. J. Obstet Gynecol Reprod Biol. 2020, 245, 13–18. 10.1016/j.ejogrb.2019.11.032. 31812702

[ref31] Moscicki A. B.; Kaul R.; Ma Y.; Scott M. E.; Daud I. I.; Bukusi E. A.; Shiboski S.; Rebbapragada A.; Huibner S.; Cohen C. R. Measurement of mucosal biomarkers in a phase 1 trial of intravaginal 3% StarPharma LTD 7013 gel (VivaGel) to assess expanded safety. J. Acquir Immune Defic Syndr 2012, 59, 134–40. 10.1097/QAI.0b013e31823f2aeb. 22067666 PMC3261360

[ref32] Mignani S.; Shi X.; Rodrigues J.; Tomas H.; Karpus A.; Majoral J. P. First-in-class and best-in-class dendrimer nanoplatforms from concept to clinic: Lessons learned moving forward. Eur. J. Med. Chem. 2021, 219, 113456 10.1016/j.ejmech.2021.113456. 33878563

[ref33] Francica J. R.; Laga R.; Lynn G. M.; Mužíková G.; Androvič L.; Aussedat B.; Walkowicz W. E.; Padhan K.; Ramirez-Valdez R. A.; Parks R.; Schmidt S. D.; Flynn B. J.; Tsybovsky Y.; Stewart-Jones G. B. E.; Saunders K. O.; Baharom F.; Petrovas C.; Haynes B. F.; Seder R. A.; Moon J. J.; et al. Star nanoparticles delivering HIV-1 peptide minimal immunogens elicit near-native envelope antibody responses in nonhuman primates. Plos Biol. 2019, 17, e3000328 10.1371/journal.pbio.3000328. 31206510 PMC6597128

[ref34] Tong W. Y.; Maira M.; Roychoudhury R.; Galan A.; Brahimi F.; Gilbert M.; Cunningham A. M.; Josephy S.; Pirvulescu I.; Moffett S.; et al. Vaccination with Tumor-Ganglioside Glycomimetics Activates a Selective Immunity that Affords Cancer Therapy. Cell Chem. Biol. 2019, 26, 1013 10.1016/j.chembiol.2019.03.018. 31105061

[ref35] Fera D.; Lee M. S.; Wiehe K.; Meyerhoff R. R.; Piai A.; Bonsignori M.; Aussedat B.; Walkowicz W. E.; Ton T.; Zhou J. O.; Danishefsky S.; Haynes B. F.; Harrison S. C.; et al. HIV envelope V3 region mimic embodies key features of a broadly neutralizing antibody lineage epitope. Nat. Commun. 2018, 9, 1111 10.1038/s41467-018-03565-6. 29549260 PMC5856820

[ref36] Williams W. B.; Meyerhoff R. R.; Edwards R. J.; Li H.; Manne K.; Nicely N. I.; Henderson R.; Zhou Y.; Janowska K.; Mansouri K.; et al. Fab-dimerized glycan-reactive antibodies are a structural category of natural antibodies. Cell 2021, 184, 2955 10.1016/j.cell.2021.04.042. 34019795 PMC8135257

[ref37] Manolova V.; Flace A.; Bauer M.; Schwarz K.; Saudan P.; Bachmann M. F. Nanoparticles target distinct dendritic cell populations according to their size. Eur. J. Immunol. 2008, 38, 1404–1413. 10.1002/eji.200737984. 18389478

[ref38] Reddy S. T.; Rehor A.; Schmoekel H. G.; Hubbell J. A.; Swartz M. A. In vivo targeting of dendritic cells in lymph nodes with poly(propylene sulfide) nanoparticles. J. Controlled Release 2006, 112, 26–34. 10.1016/j.jconrel.2006.01.006. 16529839

[ref39] Stano A.; Nembrini C.; Swartz M. A.; Hubbell J. A.; Simeoni E. Nanoparticle size influences the magnitude and quality of mucosal immune responses after intranasal immunization. Vaccine 2012, 30, 7541–7546. 10.1016/j.vaccine.2012.10.050. 23103199

[ref40] Lyu Z.; Ding L.; Huang A. Y. T.; Kao C. L.; Peng L. Poly(amidoamine) dendrimers: covalent and supramolecular synthesis. Mater. Today Chem. 2019, 13, 34–48. 10.1016/j.mtchem.2019.04.004.

[ref41] Ulbrich K.; Subr V.; Strohalm J.; Plocová D.; Jelínková M.; Ríhová B. Polymeric drugs based on conjugates of synthetic and natural macromolecules I.: Synthesis and physico-chemical characterisation. J. Controlled Release 2000, 64, 63–79. 10.1016/S0168-3659(99)00141-8. 10640646

[ref42] Tao L.; Liu J. Q.; Xu J. T.; Davis T. P. Synthesis and bioactivity of poly(HPMA)-lysozyme conjugates: the use of novel thiazolidine-2-thione coupling chemistry. Org. Biomol Chem. 2009, 7, 3481–3485. 10.1039/b907061c. 19675903

[ref43] Subr V.; Kostka L.; Strohalm J.; Etrych T.; Ulbrich K. Synthesis of Well-Defined Semitelechelic Poly[*N*-(2-hydroxypropyl)methacrylamide] Polymers with Functional Group at the α-End of the Polymer Chain by RAFT Polymerization. Macromolecules 2013, 46, 2100–2108. 10.1021/ma400042u.

[ref44] Subr V.; Konák C.; Laga R.; Ulbrich K. Coating of DNA/poly(L-lysine) complexes by covalent attachment of poly[ *N*-(2-hydroxypropyl)methacrylamide]. Biomacromolecules 2006, 7, 122–130. 10.1021/bm050524x. 16398506

[ref45] Androvic L.; Woldrichová L.; Jozefjaková K.; Pechar M.; Lynn G. M.; Kanková D.; Malinová L.; Laga R. Cyclotriphosphazene-Based Star Copolymers as Structurally Tunable Nanocarriers with Programmable Biodegradability. Macromolecules 2021, 54, 3139–3157. 10.1021/acs.macromol.0c02889.

[ref46] Neděla V.; Tihlaříková E.; Maxa J.; Imrichová K.; Bučko M.; Gemeiner P. Simulation-based optimization of thermodynamic conditions in the ESEM for dynamical *in-situ* study of spherical polyelectrolyte complex particles in their native state. Ultramicroscopy 2020, 211, 112954 10.1016/j.ultramic.2020.112954. 32018072

[ref47] Rimankova L.; Cernocka H.; Tihlarikova E.; Nedela V.; Ostatna V. Chronopotentiometric sensing of native, oligomeric, denatured and aggregated serum albumin at charged surfaces. Bioelectrochemistry 2022, 145, 108100 10.1016/j.bioelechem.2022.108100. 35334293

[ref48] Lobaz V.; Liscakova V.; Sedlak F.; Musil D.; Petrova S. L.; Sedenkova I.; Panek J.; Kucka J.; Konefal R.; Tihlarikova; et al. Tuning polymer-blood and polymer-cytoplasm membrane interactions by manipulating the architecture of poly(2-oxazoline) triblock copolymers. Colloids Surf. B Biointerfaces 2023, 231, 113564 10.1016/j.colsurfb.2023.113564. 37742364

[ref49] Tihlaříková E.; Neděla V.; Đorđević B. *In-situ* preparation of plant samples in ESEM for energy dispersive x-ray microanalysis and repetitive observation in SEM and ESEM. Sci. Rep-Uk 2019, 9, 2300 10.1038/s41598-019-38835-w. PMC6381206 30783188

