# Supplementary material for: Synthesis and Structure Optimization of Star Copolymers as Tunable Macromolecular Carriers for Minimal Immunogen Vaccine Delivery
Source: Bioconjug Chem. 2024 Jul 31;35(8):1218–32. doi: 10.1021/acs.bioconjchem.4c00273 (PMC11342300; doi:10.1021/acs.bioconjchem.4c00273)
Supplement: Supplementary file 1 — bc4c00273_si_001.pdf [file bc4c00273_si_001.pdf]

Supporting information

Synthesis and structure optimization of star copolymers as tunable macromolecular carriers for minimal immunogen vaccines delivery

Gabriela Mixová[1], Eva Tihlaříková[2], Yaling Zhu[3], Lucie Schindler[1], Ladislav Androvič[1], Lucie Kracíková[1], Eliška Hrdá[1], Bedřich Porsch[1], Michal Pechar[1], Christopher M. Garliss[3], David Wilson[3], Hugh C. Welles3, Jake Holechek[3], Qiuyin Ren[4], Geoffrey M. Lynn[3], Vilém Neděla[2], Richard Laga[1,]*

[1]Institute of Macromolecular Chemistry, Czech Academy of Sciences, Heyrovského nám. 2, 162 06 Prague, Czech Republic

[2]Institute of Scientific Instruments, Czech Academy of Sciences, Královopolská 147, 612 64 Brno, Czech Republic

[3]Barinthus Biotherapeutics North America, Inc. (formerly Avidea Technologies, Inc.), 20400 Century Boulevard, Germantown, MD, USA 20874

[4]Vaccine Research Center, National Institutes of Health, Rockville, MD, USA 20892

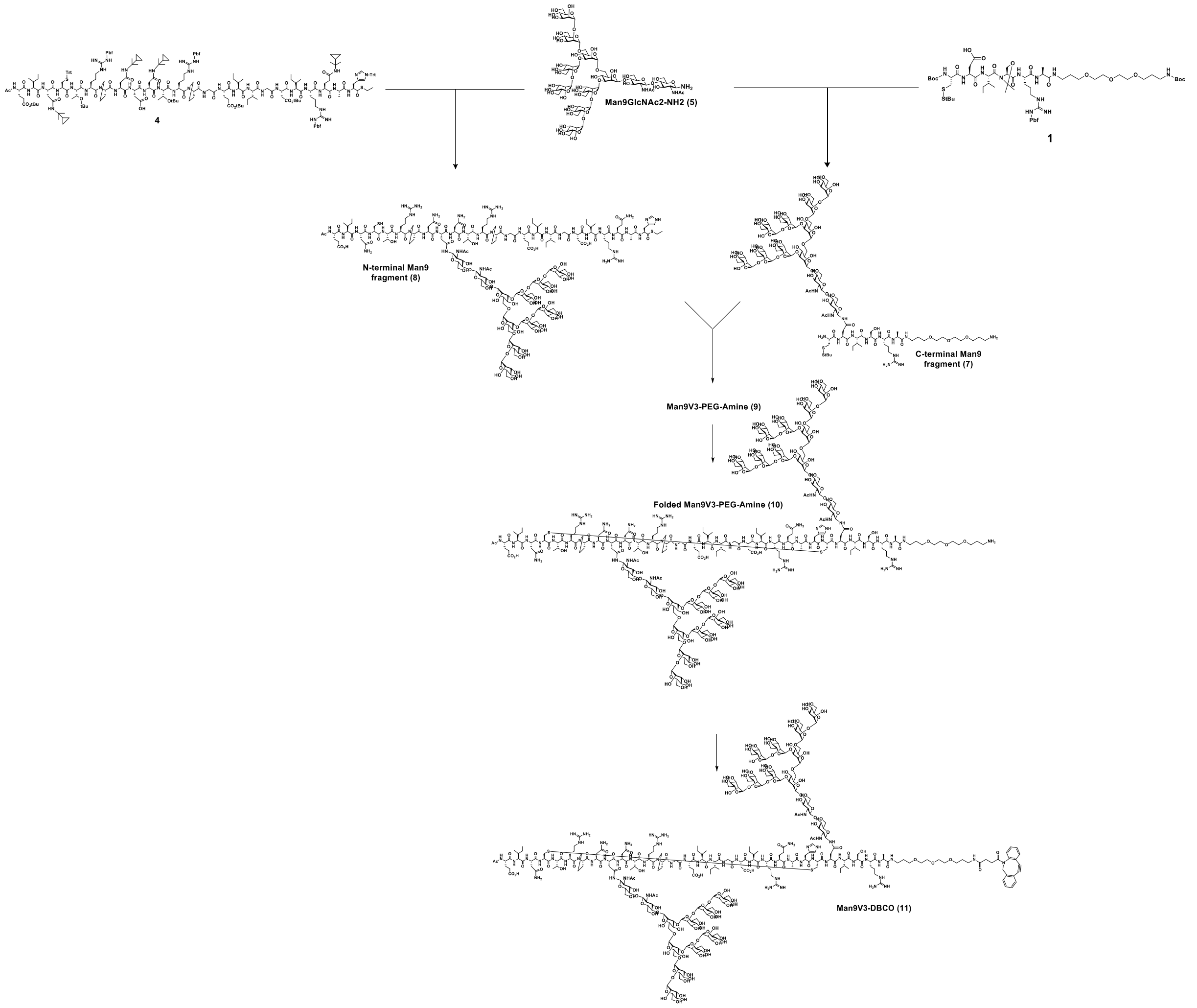

Scheme S1: Reaction scheme for the preparation of Man_9V3-DBCO.

Table S1: Characteristics of PAMAM dendrimers.

PAMAM Generation	Number of PAMAM surface NH_2 gr.	MW [kg·mol^{-1}]
**G3**	32	6.9
**G4**	64	14.2
**G5**	128	28.8

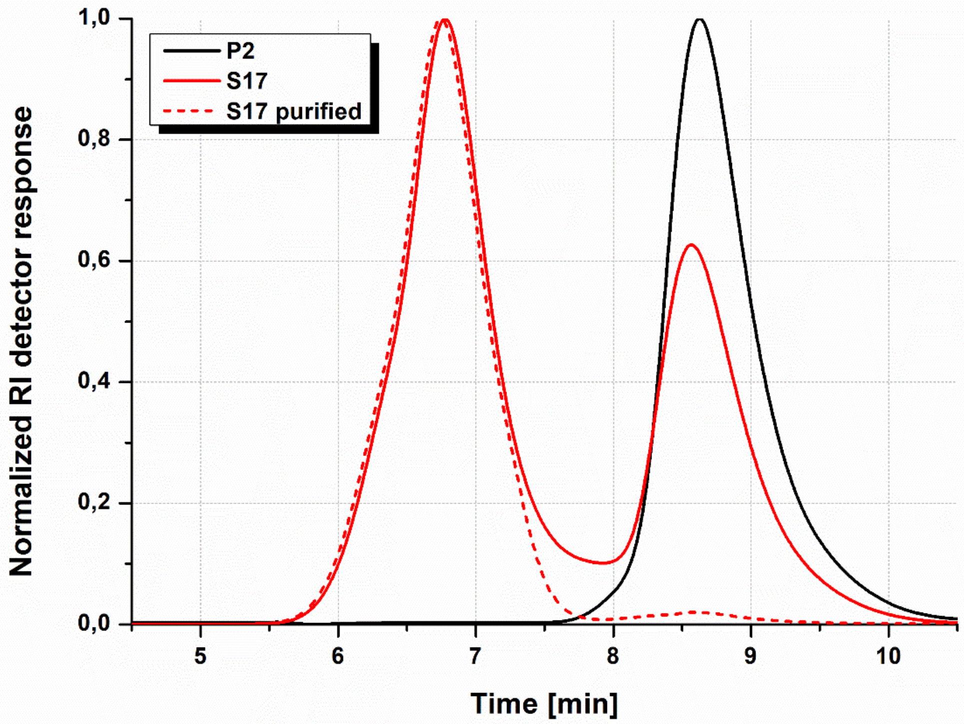

Figure S1: SEC chromatograms of the heterobifunctional polymer arm **P2**, star copolymer **S17** and purified star copolymer **S17** plotted as the normalized RI detector response as a function of retention time.

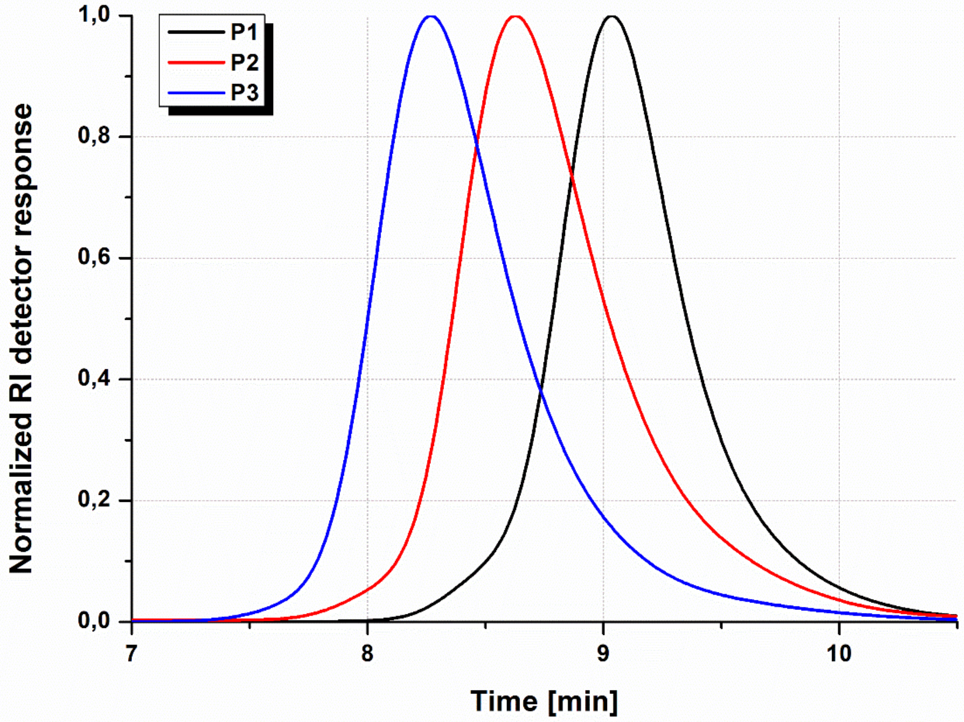

Figure S2: SEC chromatograms of the heterobifunctional polymer arms **P1–P3** plotted as the normalized RI detector response as a function of retention time.

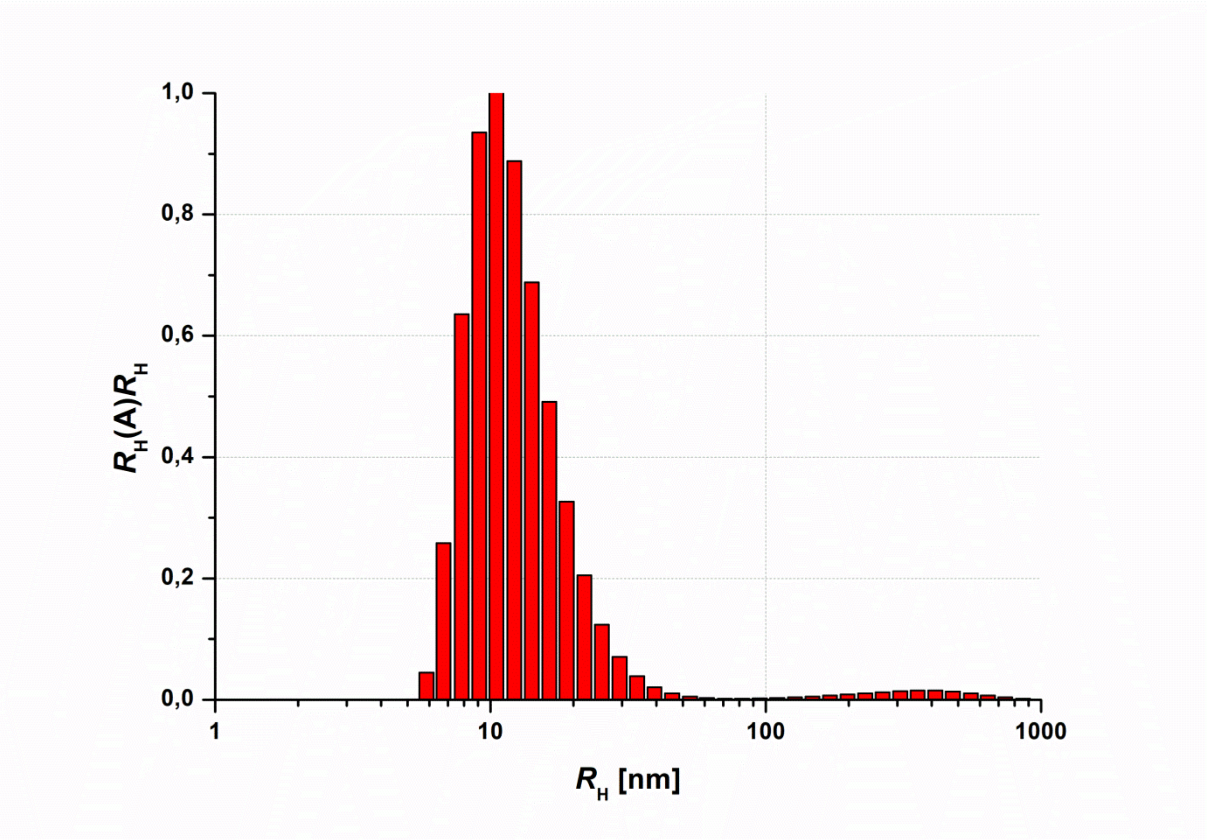

Figure S3: Normalized R_{H}-distribution function, $\mathrm{A}(R_{\mathrm{H}})$, of the purified star copolymer **S17**.

Table S2: DLS and SEC characteristics and recovery of a representative star copolymer vaccine solution (1.0 mg·mL^{-1}, PBS) before and after filtration through 0.2 µm membranes of different compositions.

Filter type	DLS	SEC			HPLC		
	R_h [i] [nm]	M_n [ii] [kg·mol^{-1}]	$Đ$ [iii]	Mass recovery [%]	Retention time [min]	AUC [mAU]	Mass recovery [%]
Non-filtered	9.0	693.2	1.04	98.0	3.477	3888.8	100.0
PTFE, 0.2 µm	9.4	629.3	1.03	96.7	3.478	3951.1	101.7
Nylon, 0.2 µm	8.5	626.8	1.03	97.0	3.480	4109.8	105.7
PES, 0.2 µm	8.9	616.9	1.03	95.6	3.479	3388.7	87.1
CA, 0.2 µm	-	-	-	-	-	-	0

[i] hydrodynamic radius of the star copolymer vaccine determined by DLS

[ii] the number-average molecular weight of the star copolymer vaccine determined by SEC

[ii] star copolymer vaccine dispersity defined as the ratio of weight-average (M_w) to number-average (M_n) molecular weight determined by SEC

Table S3: DLS and SEC characteristics and mass recovery of a representative star copolymer (A) and star copolymer vaccine (B) incubated in PBS solution (1.0 mg·mL^{-1}) at various temperatures for up to 16 weeks.

A

**Condition**	**DLS**	**SEC**		
	R_h[i] [nm]	M_n[ii] [kg·mol^{-1}]	$Đ$[iii]	Mass recovery [%]
37 ˚C in PBS, 0 days	9.0	484.0	1.07	98.0
37 ˚C in PBS, 2 days	6.8	444.0	1.05	95.1
37 ˚C in PBS, 5 days	8.5	434.3	1.05	93.6
37 ˚C in PBS, 2 weeks	7.0	392.6	1.06	90.0
37 ˚C in PBS, 4 weeks	7.0	342.6	1.04	84.2
37 ˚C in PBS, 16 weeks	4.6	169.9	1.10	58.8

B

**Condition**	**DLS**	**SEC**		
	R_h[i] [nm]	M_n[ii] [kg·mol^{-1}]	$Đ$[iii]	Mass recovery [%]
25 °C in PBS, 0 days	10.0	679.8	1.01	96.5
25 °C in PBS, 5 days	10.5	694.2	1.06	93.6
4°C in PBS, 5 days	12.3	680.0	1.05	96.3
4°C in PBS, 4 weeks	10.6	714.9	1.04	96.1
4°C in PBS, 16 weeks	8.9	735.1	1.09	98.0
–20 °C in PBS, 16 weeks	8.3	662.1	1.04	96.4
25 °C lyophilized, 5 days	10.7	709.4	1.05	96.7
4 °C lyophilized, 5 days	9.9	687.2	1.04	96.9
4 °C lyophilized, 4 weeks	13.1	700.7	1.04	96.1
4 °C lyophilized, 16 weeks	9.3	662.3	1.04	95.0
–20 °C lyophilized, 16 weeks	8.9	649.5	1.04	96.2

[i] hydrodynamic radius of the star copolymer/star copolymer vaccine determined by DLS

[ii] the number-average molecular weight of the star copolymer/star copolymer vaccine determined by SEC

[ii] star copolymer/star copolymer vaccine dispersity defined as the ratio of weight-average (M_w) to number-average (M_n) molecular weight determined by SEC

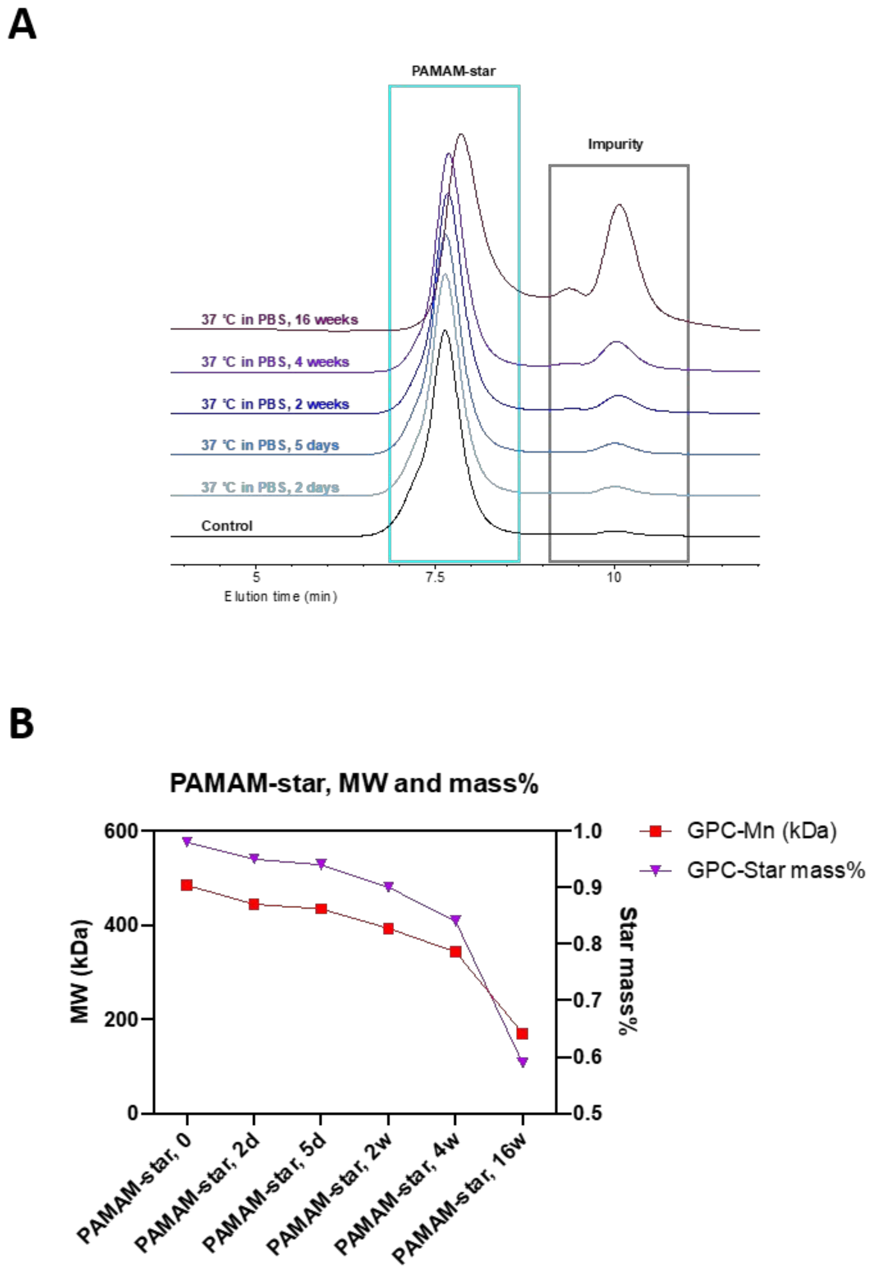

Figure S4: Temporal stability of star copolymer incubated in PBS buffer at 37 °C evaluated from change in molecular weight and injected mass. (**A**) SEC chromatograms of star copolymer injected at various time points (2 days–16 weeks) plotted as normalized RI detector response over time. (**B**) Evolution of molecular weight (M_n) and injected mass of star copolymer at various time points (2 days–16 weeks).

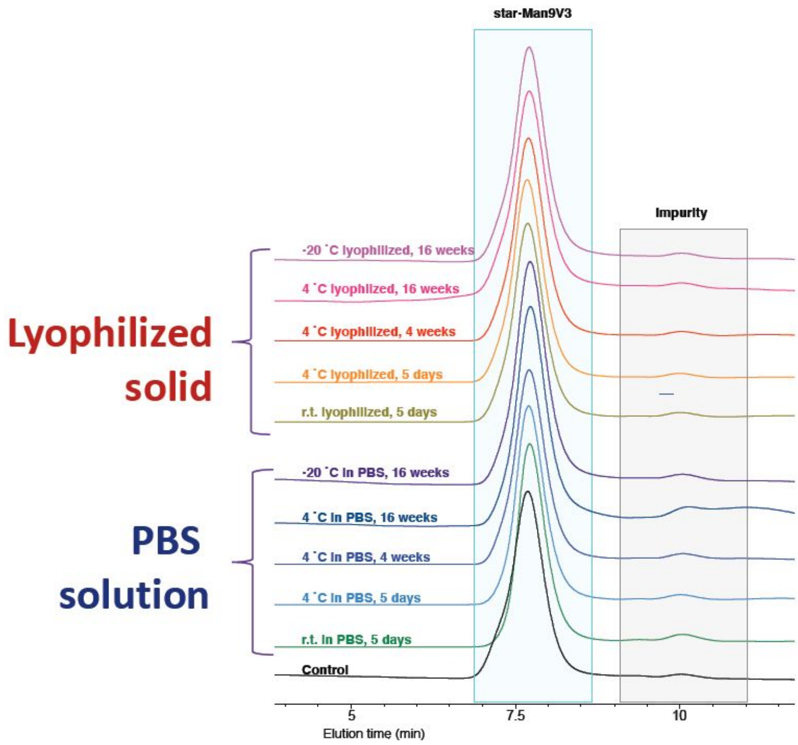

Figure S5: Stability of star copolymer vaccines stored under various conditions, including 25 °C for 5 days and 4 °C and –20 °C for up to 16 weeks either as a lyophilized solid or in PBS solution evaluated from change in molecular weight. Figure represents SEC chromatograms of injected star copolymer vaccine plotted as normalized RI detector response over time.
